# Research progress of abnormal lactate metabolism and lactate modification in immunotherapy of hepatocellular carcinoma

**DOI:** 10.3389/fonc.2022.1063423

**Published:** 2023-01-06

**Authors:** Yiwei Xu, Xiaodong Hao, Yidan Ren, Qinchen Xu, Xiaoyan Liu, Shuliang Song, Yunshan Wang

**Affiliations:** ^1^ Marine College, Shandong University, Weihai, China; ^2^ Department of Clinical Laboratory, The Second Hospital, Cheeloo College of Medicine, Shandong University, Jinan, Shandong, China; ^3^ Department of Clinical Laboratory, Shandong Provincial Hospital Affiliated to Shandong First Medical University, Jinan, Shandong, China

**Keywords:** lactate metabolism, lactylation, immune regulation, immunotherapy, hepatocellular carcinoma

## Abstract

Tumors meet their energy, biosynthesis, and redox demands through metabolic reprogramming. This metabolic abnormality results in elevated levels of metabolites, particularly lactate, in the tumor microenvironment. Immune cell reprogramming and cellular plasticity mediated by lactate and lactylation increase immunosuppression in the tumor microenvironment and are emerging as key factors in regulating tumor development, metastasis, and the effectiveness of immunotherapies such as immune checkpoint inhibitors. Reprogramming of glucose metabolism and the “Warburg effect” in hepatocellular carcinoma (HCC) lead to the massive production and accumulation of lactate, so lactate modification in tumor tissue is likely to be abnormal as well. This article reviews the immune regulation of abnormal lactate metabolism and lactate modification in hepatocellular carcinoma and the therapeutic strategy of targeting lactate-immunotherapy, which will help to better guide the medication and treatment of patients with hepatocellular carcinoma.

## Introduction

Lactate has previously been mistakenly thought to be a metabolic waste product of glycolysis under hypoxic conditions. However, the lactate shuttle hypothesis proposed by Brooks in 1985 describes the role of lactate as fuel to coordinate systemic metabolism and as signaling molecules to coordinate signaling between different cells, tissues, and organs (1, 2). As an important carbon source for cell metabolism, lactate is involved in carbon cycling *in vivo*, and is also an important signaling molecule in inflammatory and cancerous tissues (3). Cancer cells metabolize glucose differently than normal cells. Normal cells produce large amounts of lactate only under hypoxic conditions, whereas tumor cells tend to convert pyruvate to lactate even when sufficient oxygen is present through the mitochondrial TCA cycle to support oxidative phosphorylation to generate ATP, the process is an aerobic glycolysis, also known as the “Warburg effect” (4, 5). Lactylation is a novel post-translational modification (PTM) that includes histone lactylation and non-histone lactylation (6–9). The large amount of lactate produced by tumor tissue through aerobic glycolysis provides a substrate for lactylation. The biological function of lactylation in a range of diseases and cancers is being investigated.

The occurrence, metastasis, invasion and drug resistance of hepatocellular carcinoma (HCC) are largely influenced by the tumor microenvironment, which contains complex interactions between various immune or non-immune cells (10–14). Lactate has emerged as a key regulator in maintaining cancer initiation, progression, and immune escape, and lactate accumulation and lactylation in HCC enhance the immunosuppressive properties of tumor microenvironment(TME). The lactate metabolic crosstalk in the TME may be an important factor affecting the progression, immunotherapy and prognosis of HCC. By targeting lactate metabolism and restoring the metabolic adaptability of host anti-tumor immunity, the therapeutic effect of cancer immune checkpoint blockade can be further improved. Therefore, anti-lactate therapy combined with immunotherapy has broad prospects.

## Reprogramming of glucose metabolism in HCC promotes lactate production

Metabolic associated fatty liver (MAFLD) has been proposed as a more appropriate new nomenclature for nonalcoholic fatty liver disease (NAFLD) that more accurately reflects the drivers of the disease (15). Altered liver metabolism is critical to the development of liver disease, contributing to the progression of NAFLD and nonalcoholic steatohepatitis (NASH), which progressively become major risk factors for hepatocellular carcinoma (16). Lactate levels in the blood and liver also increase with the severity of liver disease, particularly from steatosis to NASH (17, 18). HCC cells are metabolically different from normal hepatocytes. In order to meet the demands of tumor tissue for energy, redox, and biosynthesis, HCC cells exhibit active anaerobic glycolysis and aerobic glycolysis (Warburg effect). In addition, metabolism-related enzymes and transport proteins are reprogrammed (19, 20). Upregulated glucose transporters (GLUTs) promote glucose uptake, increased expression of hexokinase (HK), phosphofructokinase 1 (PFK1) and pyruvate kinase (PKM) accelerates glycolysis, and overexpression of lactate dehydrogenase (LDH) drives the rapid conversion of pyruvate to lactate (21). Ultimately one of the results of this metabolic reprogramming is the production of large amounts of lactate.

## Production of lactate

**Figure 1:**
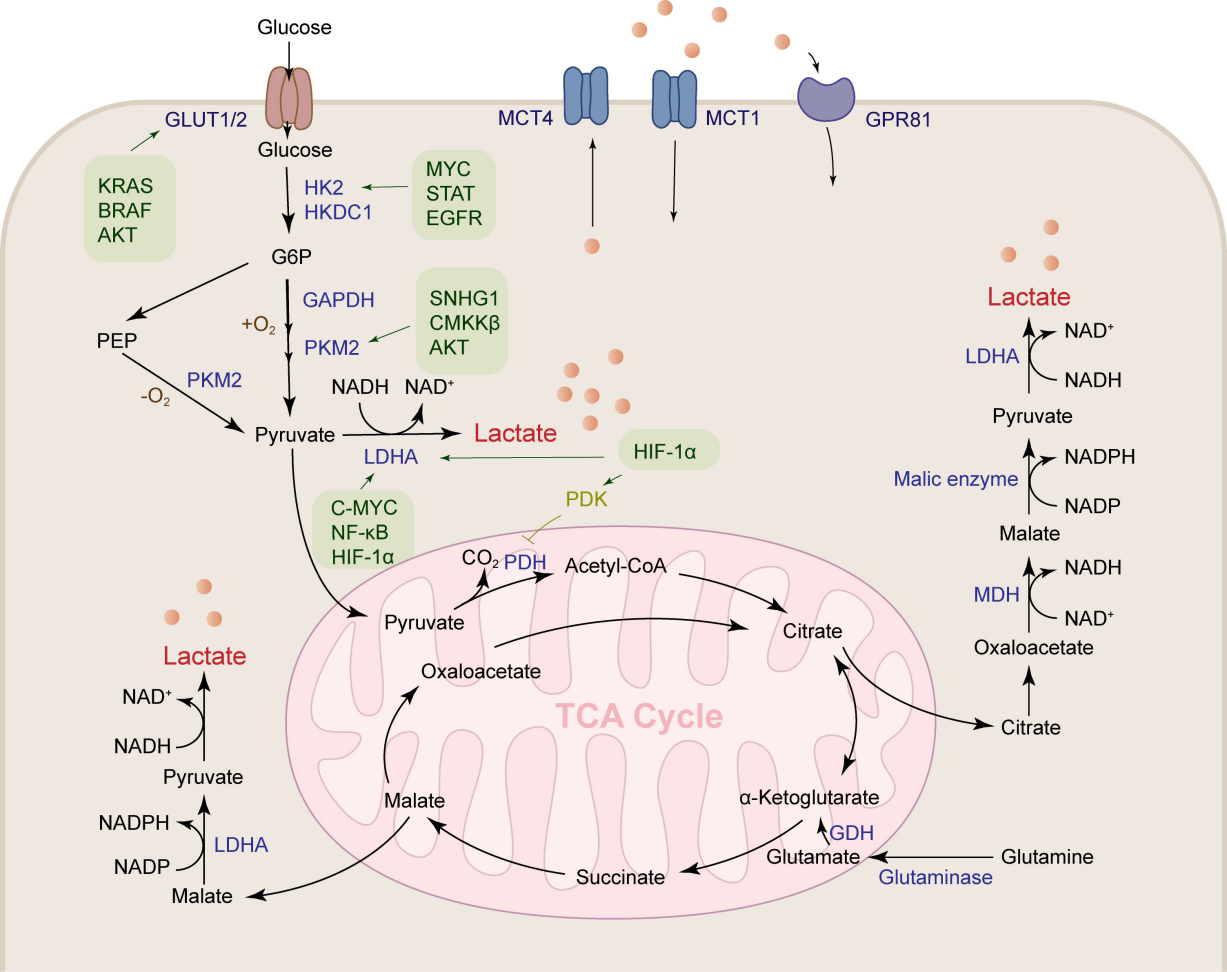
Lactate is produced by a variety of pathways. Lactates are mainly produced by the aerobic glycolysis pathway and the glutamine pathway. Glucose metabolism reprogramming in HCC promotes glucose uptake by the glucose transporter GLUT1/2. HK2, GAPDH, PKM2 and LDHA are upregulated under the regulation of a variety of cytokines to accelerate lactate production. Glutamine is transported into mitochondria, where it produces α-ketoglutaric acid under the action of GDH, followed by participation in the TCA cycle to produce malate, and finally transported out of the mitochondria to produce lactate.

Lactate is produced through abundant pathways in tumor tissue, such as pentose phosphate pathway, malate-mediated glutamine pathway, and citrate-mediated pyruvate production, which ultimately lead to the secretion of lactate salts (**Figure 1**). Glucose is transported into cancer cells by GLUT1/3 transporters and then undergoes glycolysis to produce pyruvate. Under positive oxygen conditions, pyruvate is converted to acetyl-CoA by pyruvate dehydrogenase and releases carbon dioxide (CO2), which enters the tricarboxylic acid (TCA) cycle. Under anaerobic conditions, glucose is metabolized to pyruvate by phosphoenolpyruvate (PEP), which is then converted to lactate by lactate dehydrogenase A (LDHA). Tumor cells meet the energy requirements for growth and proliferation of cancer cells by increasing glycolysis, while glucose is metabolized at a higher rate by aerobic glycolysis (Warburg effect) (5). Compared with lactate produced by complete oxidation of glucose in mitochondria, tumor tissue produces 10-100 times more lactate through aerobic glycolysis (22).

Glucose is the main metabolic substrate for lactate production, and glutamine is also an important substrate for lactate production. Tumor cells have a clear Warburg effect, which is enough to support most of the requirements of the cell for ATP synthesis, and the construction of lipids, proteins, and nucleotides required for cell growth mainly comes from the TCA cycle. But in these cells, their TCA cycle is intact, and this requirement is met by high glutamine metabolism. They can utilize the carbon backbone of glutamine as a respiratory substrate, activate the NADPH-producing pathway, and restore oxaloacetate to continue TCA cycle function (23, 24). Continued glutamine metabolism provides an intermediate for the TCA cycle and also makes aerobic glycolysis the best choice for cancer growth (25). Glioblastomas, for example, convert about 90 percent of glucose and 60 percent of glutamine to lactate (24). Glutamine is converted to glutamate by glutaminase, glutamate is converted to α-ketoglutarate by glutamate dehydrogenase (GDH) in mitochondria, and then α-ketoglutarate is converted to malate and then transported out of the mitochondria, oxidized to pyruvate in the cell matrix, and finally lactate is produced under the action of LDHA (26). Glutamine can also provide carbon in the form of mitochondrial oxaloacetate to generate citrate in the first step of the TCA cycle, mediating lactate and lipid biosynthesis. Glutamine also provides a carbon source to support the TCA cycle during glucose starvation (27).

In addition to the above methods, activated immune cells can also metabolize lactate, such as dendritic cells (DC) depending on the Warburg effect (28). CD28-stimulated T cells rapidly increase the expression of glucose transporters and the rate of glycolysis (29, 30). In macrophages, monocarboxylate transporter 4 (MCT4) is upregulated by the stimulation of TLR2 and TLR4, activating an inflammatory response to increase the rate of glycolysis to produce lactate (31). But they contribute less to lactate in the tumor microenvironment because it depends on the number of immune cells in the tumor microenvironment and their activation status. In conclusion, the accumulation of lactate in HCC tumor tissue is largely due to its active production pathway.

## Metabolic reprogramming promotes lactate production in HCC

To meet the demands for energy and biosynthesis, HCC tumor tissues metabolically reprogram key enzymes of their glycolysis. HCC tumors enhance glucose uptake by upregulating the glucose transporters GLUT1 and GLUT2 (32, 33), and HIF-1 is an important accelerator of this process (34). In addition, KRAS mutation, BRAF mutation and activated AKT cascade increased GLUT1 expression (35, 36).

Glucose is converted into glucose-6-phosphate by hexokinase (HK) after entering cells. Among HK family members, HK2 and HKDC1 are up-regulated in HCC and are associated with poor prognosis (37, 38). Silencing HK2 and HKDC1 inhibited lactate flux, inhibited HCC cell proliferation and migration *in vitro*, increased oxidative phosphorylation, and metformin sensitivity (38, 39). The expression of HK2 is induced in HCC by multiple mechanisms and oncogenic transcription factors. HK2 was recently identified as a downstream target of ZMYND8 in HCC cells, and high expression of ZMYND8 in HCC was associated with glucose consumption, increased lactate, and ATP production in HCC cells, and was associated with patients with unfavorable clinicopathological features and poor prognosis. Silencing ZMYND8 inhibited the proliferation and migration of HCCLM3 cells *in vivo (* 40). MACC1 and STAT3 also enhance glucose metabolism and lactate production through HK2 (41, 42). Blockade of FOXK1, EGFR, C-MYC signaling inhibits HK2-mediated glycolysis (42–44).

Glyceraldehyde-3-phosphate dehydrogenase (GAPDH) has been considered as a stably expressed reference gene in the past, and GAPDH has been reported to be up-regulated in various cancers (45). The interaction of GAPDH with hepatitis viruses (HBV and HCV) induces hepatitis, as well as metabolically enhances glycolytic capacity (46, 47).

The pyruvate kinase isoform PKL is an important enzyme involved in the final step of glycolysis in normal hepatocytes, while pyruvate kinase 2 (PKM2) is overexpressed in HCC cells and is an independent predictor of recurrence and survival (48, 49). As an active protein kinase, PKM2 promotes hepatocellular carcinoma cell proliferation by upregulating HIF-1α, Bcl-xl and Gli1 expression (50). PKM2 plays a synergistic effect with ODC1, which is an important enzyme involved in polyamine metabolism (51), and is also affected by various signals such as SNHG1 and CaMKKβ, as well as the AKT pathway (52–54). Genes related to polyamine metabolism are overexpressed in HCC patients, which also links polyamine metabolism to abnormal lactate metabolism promoting poor prognosis. Decreased expression of PKM2 inhibits glucose uptake by HCC cells and inhibits aerobic glycolysis (48). Altered expression of the aforementioned enzymes supports glucose flux in the glycolytic pathway, leading to an increase in the end product of glycolysis, pyruvate, which is available for the TCA cycle as well as lactate production.

The Warburg effect is a key event in hepatocarcinogenesis, where pyruvate tends to be converted to lactate catalyzed by lactate dehydrogenase (LDH) even under aerobic conditions. In hepatocellular carcinoma cells, LDHA is overexpressed due to downregulation of miR-383, triggering increased cell proliferation, invasion and glycolysis (55), MYC, NFκB, HIF-1α-mediated signaling enhances glycolysis in HCC by promoting upregulation of LDHA (56–58). In contrast, knockdown of LDHA significantly inhibited tumor growth and metastasis of hepatocellular carcinoma as well as the Warburg-like metabolic signature of mouse HCC (59, 60). LDH levels in serum have been regarded as a prognostic indicator in HCC patients treated with sorafenib, transarterial-chemoembolization (TACE), and partial hepatectomy (61–63). In addition to these enzymes as important regulators of the Warburg effect, the Warburg effect is also regulated by other complex mechanisms, such as the transcriptional activation of PFKM by ZEB1 and the direct targeting of FBP1 by miR-517a to enhance the Warburg effect of liver cancer (64, 65); PGC1α inhibits the Warburg effect by regulating the WNT/β-catenin/PDK1 axis (66).

## Transport of lactate

Lactate is mainly transported by the monocarboxylate transporter (MCT) on the cell membrane, MCT1 (SLC16A1) mainly imports lactate, and MCT4 (SLC16A3) mainly exports lactate. MCT1 can also mediate lactate export under hypoxic conditions (67–69). It has been reported that MCT4 is highly expressed in HCC and promotes tumor progression (70), and inhibition of CD147 or MCT1 inhibits lactate export and glucose metabolism, and inhibits HCC proliferation (71). The poor prognosis of multiple types of cancers is associated with high expression of MCT1 and MCT4, such as glioma (72), breast cancer (73), non-small cell lung cancer (74), colorectal cancer (75), gastric cancer (76), cervical cancer (77) and neuroblastoma (78).

G protein-coupled receptor 81 (GPR81) is a lactate-selective receptor that is highly expressed in many tumor cell lines, such as breast, colon, lung, hepatocellular, salivary gland, cervical, and pancreatic cancers. The expression level of GPR81 affects tumor growth and metastasis, and knockdown of GPR81 results in significantly reduced growth and metastasis of pancreatic cancer cells and breast cancer cells (79, 80). Inhibition of GPR81 signaling and thus angiogenesis is mediated by PI3K/AKT-cAMP in response to CREB (81). Activation of GPR81 aggravates hepatic ischemia-reperfusion injury-induced remote organ injury (82).This suggests that GPR81 is essential for cancer cells to regulate lactate transport, tumor growth and metastasis, angiogenesis, and lipolysis inhibition (79).

## Lactate homeostasis and metabolic abnormalities

Cells in humans typically have lactate concentrations of 1-3 mM at rest, and transiently increase to 15 mM during exercise, however, in highly glycolytically active tumor cells, lactate concentrations even reach 30-40 mM (83). Lactate is not only a substrate for glycolysis, but also the main fuel for maintaining the carbon cycle, and is rapidly exchanged in the body to provide an energy substrate for cellular metabolism. Studies have shown that, in addition to the brain, the contribution of glucose to the tissue TCA cycle is indirect, but mainly through circulating lactate. In genetically engineered lung and pancreatic cancer tumors in fasted mice, lactate was the main source of carbon for the TCA cycle (84). In addition, lactate and pyruvate together act as a circulating redox buffer, balancing the NADH/NAD ratio (85). Lactate is also reused by different cell subsets in the TME, a phenomenon known as metabolic symbiosis (86). The function of lactate depends on its concentration in the organism, and the normal production and transport of lactate is the basis for maintaining the lactate cycle in the body. Under the coordinated action of oncogenes and tumor suppressors, tumor cells produce a large amount of lactate through aerobic glycolysis, local TME acidification, and the homeostasis of lactate is severely disrupted in the TME. HIF-1α, c-MYC, PI3K/AKT increase glycolytic flux by increasing the expression of glucose transporter, hexokinase, phosphofructokinase (26). Pyruvate dehydrokinase (PDK) inhibits the activation of pyruvate dehydrogenase (PDH) by phosphorylation, preventing pyruvate from entering the mitochondria and being converted to acetyl-CoA for the TCA cycle. Hypoxia-inducible factor 1α (HIF-1α) stimulates the expression of PDK and LDHA, resulting in the conversion of pyruvate to lactate (87). C-MYC is an oncogene that stimulates glycolysis as well as the expression of LDHA. LDHA is one of the key enzymes in the conversion of glucose and glutamine to lactate. Increased activity of LDHA, increased glycolysis, and increased production of lactate, inhibiting LDHA activity affects cancer cell proliferation (88).

**Figure 2:**
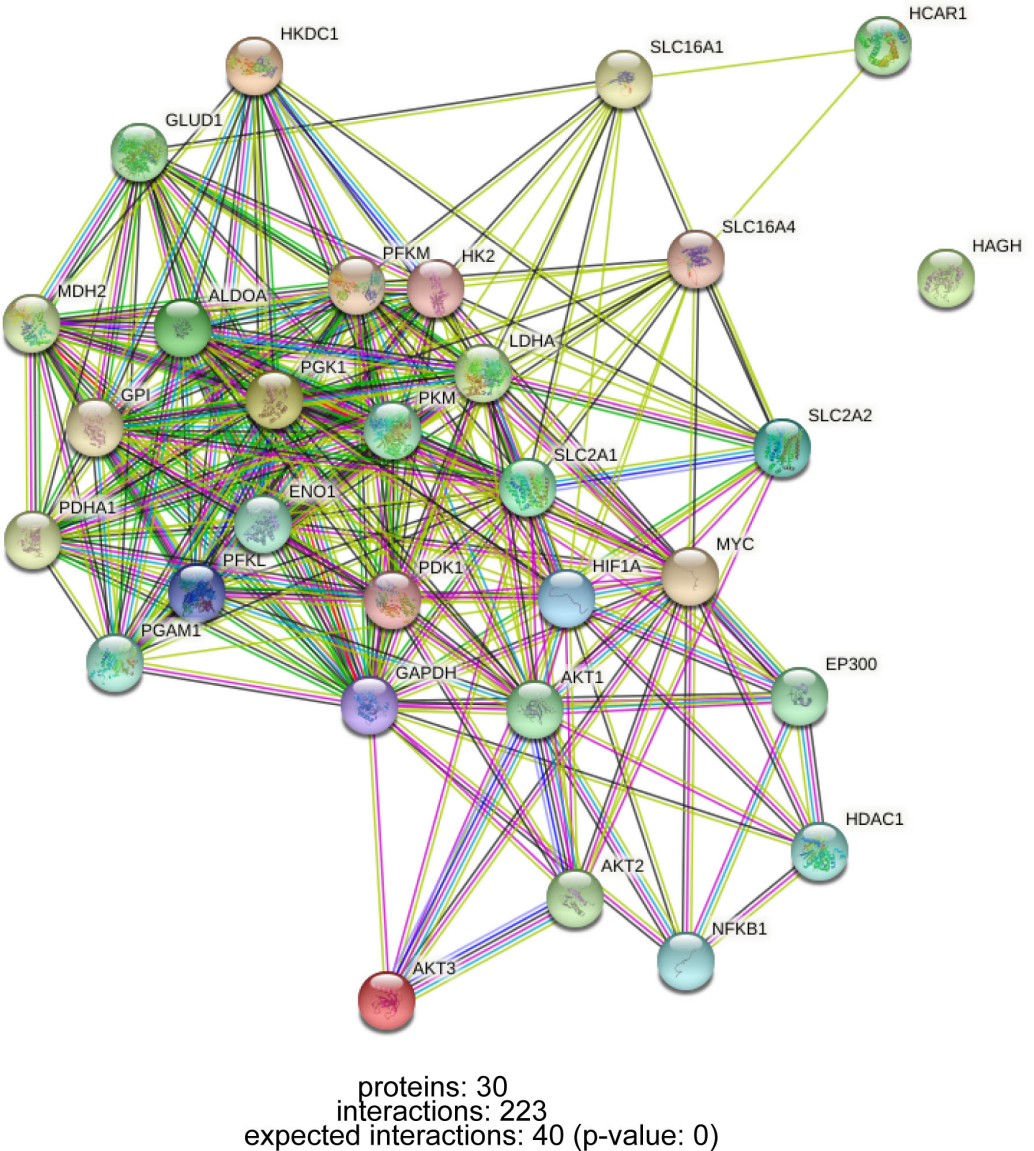
PPI network of interactions between lactate metabolism-related genes.

Lactate accumulation in tumor tissue is a combined result of increased production and decreased clearance. The net clearance of lactate by the healthy liver accounts for 70% of the systemic clearance, showing higher clearance than other organs (89). P300/CBP-associated factor (PCAF)-mediated acetylation of LDHB reduces LDHB activity and inhibits lactate clearance, leading to lactate accumulation, which exacerbates lipid deposition and inflammatory responses in NAFLD and NAFLD progression (90). In conclusion, the abnormal metabolism of lactate is regulated by a complex network of genes. In addition to genes that directly regulate glucose metabolism and lactate formation, oncogenes and tumor suppressor genes such as HIF-1 and MYC are also involved in glucose metabolism reprogramming during carcinogenesis (21), the interaction of these genes is shown in **Figure 2**.

## Lactylation of HCC

### Histone lactylation

**Figure 3:**
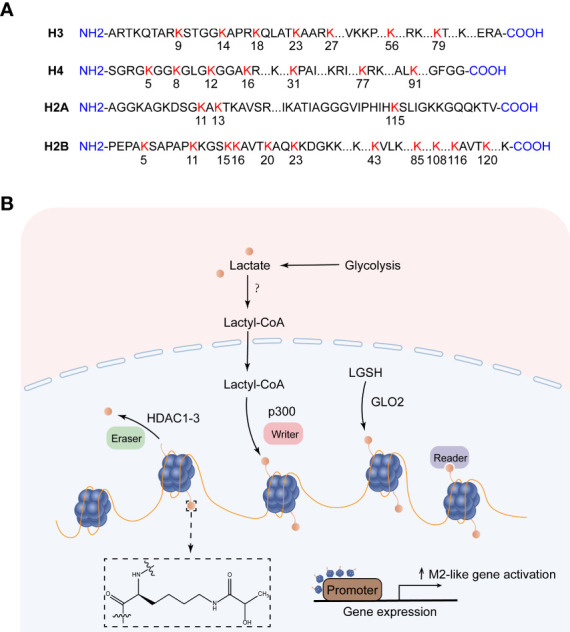
Histone lysine lactylation site and the production of lactylation. **(A)** Histone lysine lactylation site. **(B)** Lactate metabolism can induce epigenetic remodeling through histone lactylation. After lactate produces lactyl-CoA, the lactosyl group is transferred by p300 to the lysine tail of the histone protein, forming a lactylation modification. Lactyl glutathione (LGSH) hydrolyzed to produce lactate, which forms lactylation modifications through non-enzymatic reactions. HDAC1-3 is a potential delactinase. It is unclear which enzymes produce lactyl-CoA and which enzymes recognize histone lactylation.

Post-translational modifications of histones, such as methylation, acetylation, phosphorylation, and ubiquitination, maintain homeostasis by regulating DNA transcription, replication, and repair, and their misregulation is closely related to the occurrence and development of many diseases (91). With the application of high-sensitivity mass spectrometry, some new short-chain Lys acylations of histones have been discovered, such as propionylation (Kpr), butyrylation (Kbu), 2-hydroxyisobutyrylation (Khib), Succinylation (Ksucc) et al (92). Zhang et al. first predicted and identified lysine lactylation (Kla) as a novel histone modification stimulated by endogenous lactate. They used MS/MS analysis to identify 26 and 16 histone Kla sites from human MCF-7 cells and mouse bone marrow-derived macrophages (BMDM), respectively (9)(**Figure 3A**). Yang et al. identified 27 histone Kla sites from gastric cancer AGS cells (8). Mouse cancer models show that the expression of the M2-like gene Arg1 is positively correlated with histone Kla levels. Zhang et al. knocked down LDHA or used glycolysis inhibitors during M1 polarization of macrophages induced by lipopolysaccharide (LPS) and interferon gamma (IFN-γ). The results showed decreased lactate production as well as global histone Kla levels, decreased ARG1 expression, and decreased histone Kla levels at the ARG1 promoter (9). Stefanie Dichtl et al. also confirmed that the expression of ARG1 in LPS-stimulated cells was mediated by IL-6, and the increase of ARG1 was dependent on the increase of lactate levels (93). The study identified four pathways of increased histone lactylation (1): Increased glucose to increase glycolysis; (2) Rotenone, an inhibitor of the mitochondrial respiratory chain complex I, drives glycolysis; (3) Hypoxia; (4) M1 macrophage polarization (9).

So how does lactate mediate histone lactylation modification, and how does histone lactylation change the transcriptional landscape? Existing studies have shown that lactoyl-CoA produced by endogenous or exogenous (mostly endogenous) lactate, the acetyltransferase p300 acts as a writer to transfer the lactosyl group to the lysine tails of histones to create a modification called lactylation (9). Both lactylation and acetylation prefer lysine (Lys) as a residue of epigenetic modifications, and they compete for the same enzyme p300. However, how these enzymes decide which epigenetic modification to encode in histones remains a mystery, possibly regulated by differential recruitment of cofactors in response to cellular metabolic dynamics (94). In addition to the enzymatic transfer of lactyl-CoA to lysine, lactyl-glutathione (LGSH) is hydrolyzed by glyoxalase 2 (GLO2) to generate glutathione and D-lactate, the lactate moiety is nonenzymatically transferred from LGSH to lysine residues to form lactylation modifications (95). Recent studies have shown that Class I histone deacetylases (HDAC1-3) act as erasers to exhibit delactylase activity *in vitro (* 96, 97), Sirtuins are potential non-histone delactate enzymes (98). However, it is not clear which enzymes produce the intermediate molecule Lactyl-CoA, which enzymes recognize histone lactylation as “readers”, and more “writers” and “eraser” are yet to be discovered (**Figure 3B**). ChIP-seq data showed that H3K18la, like H3K18ac, was enriched in the promoter region (± 2 kb around the transcription start site) and indicated steady-state mRNA levels. In contrast to H3K18ac, the increased H3K18la marked more genes than the decreased H3K18la, and most genes marked by elevated H3K18la were specific. Zhang et al. took advantage of a cell-free, recombinant chromatin-templated histone modification and transcription assay, and they demonstrated that histone Kla plays a direct role in transcriptional regulation. In this assay, acetyl-CoA is replaced by L-lactyl-CoA, showing strong p53-dependence, p300-mediated H3 and H4 lactate salting, and effects on transcription. The H3 and H4 mutations eliminate p300 and p53-dependent transcription. These findings suggest that transcription is directly mediated by histone lactylation rather than mediating other transcription factors in the nucleus (9). Furthermore, H3K18la is enriched not only at the promoter but also at the active enhancer in a tissue-specific manner (99). Eva Galle et al. calculated ChromHMM state enrichment over ENCODE’s database of cell type agnostic candidate cis-regulatory elements (cCRE). They found that each ChromHMM state enriched with a distal enhancer-like sequence (dELS) was always marked with H3K18la. H3K18la marks active promoters and active enhancers, which are typically marked by H3K27ac (typical mark of active promoters and activity enhancers). And a considerable part of the presumed dELS only H3K18la peak marker, but not H3K27ac peak marker, indicating that dELS has additional H3K18la-specific effects. Research on histone lactylation is still in its infancy, and delving into how this post-translational modification affects the transcriptional landscape will broaden our horizons in the treatment of disease (99).

Current studies have demonstrated that various disease states are regulated by histone lactylation. Increased histone lactylation correlates with inflammation-induced macrophage death. Phosphoinositide 3-kinase (BCAP) promotes the transition of macrophages from an inflammatory state to a repair state through histone lactylation in response to microbial ligands and various deleterious signals (100). Lung myofibroblasts promote the fibrotic activity of macrophages through lactate-induced histone lactylation in macrophage profibrotic gene promoters (101). A clinical study by Chu et al. showed that histone H3K18 lactylation aggravated the severity of septic shock in patients (102). Macrophages can take up lactate through MCT and promote HMGB1 lactylation through the p300/CBP pathway. Inhibiting lactate levels *in vivo* or inhibiting lactate signaling can reduce exosomal HMGB1 lactylation levels, thereby ameliorating multiple microbial sepsis (103). Yang et al. further identified the lactylation modification site HMGB1-K43la in HMGB1 (8). Glis1 acts as a reprogramming factor to promote somatic reprogramming by enhancing histone acetylation (H3K27ac) and lactylation (H3K18la) at pluripotent gene loci (104). Histone Kla is also widely distributed in the brain, and the level of histone H1Kla in the brain increases in response to the expression of the neuronal activity marker c-Fos and the neural excitation induced by Repeated social defeat stress (SDS) (105). H3K18la is involved in remodeling transcriptome expression and activates transcription in brain neurons (97).

Cancer cells produce more lactate than normal cells through the “Warburg effect”, so it is likely that histone lactylation in tumors is abnormal (106) (**Table 1**). Yu et al. found for the first time that the level of histone lactylation was significantly up-regulated in ocular melanoma, and inhibition of histone lactylation could effectively inhibit tumor progression. Their study found that histone lactylation promotes YTHDF2 expression in ocular melanoma, and that YTHDF2 binds to m^6^A sites on the mRNAs of PER1 and TP53 to mediate RNA degradation, thereby driving carcinogenesis (107). Lactate in the TME induces METTL3 expression in tumor-infiltrating myeloid cells (TIMs) through histone lysine K18 lactylation, METTL3 lysine K281 and K235 lactylation-mediated RNA m6A modification leads to tumor immune escape by promoting the immunosuppressive function of TIM (108). Disturbed lactate metabolism in non-small cell lung cancer (NSCLC) mediates the expression of genes such as HK-1 and IDH3G through histone lactylation, regulating mitochondrial homeostasis as well as cellular metabolism (109). Inactive von Hippel-Lindau (VHL) is an important factor in the pathogenesis of clear cell renal cell carcinoma (ccRCC), which exerts oncogenic effects by inducing histone lactylation to activate platelet-derived growth factor receptor beta (PDGFRβ) expression. In turn, PDGFRβ positively feedback regulates histone lactylation (110). Histone lactylation at the promoter of Gram-negative bacteria-derived lipopolysaccharide in colorectal tumor tissues reduces the binding efficiency of the inhibitory factor YY1, resulting in the overexpression of LINC00152 to promote colorectal cancer cell migration and invasion (97).

**Table 1:** Lactylation in Disease.

Disease	Modification site	Cell	Protein targets	Gene targets	Reference
Lung fibrosis	Lysine	Macrophages			(101)
Septic shock	H3K18 HMGB1-K43	Macrophages			(8, 102, 103)
Ocular melanoma	K3K18		YTHDF2	m^6^A	(107)
Colon cancer	H3K18	TIMs	METTL3	m^6^A	(108)
NSCLC	Lysine		HK-1 IDH3G		(109)
ccRCC	Lysine	Macrophages	PDGFRβ		(110)
HCC HCC	MOESIN-Lys72 H3K9la, H3K56la	Tregs			(111) (112)

Lactate can induce the expression of liver injury-related genes, leading to acute liver failure (113). Accumulation of lactate is responsible for histone lactylation in inflammation and cancer. The formation of histone lactylation modification mainly depends on the enzymatic transfer of lactyl-CoA, Varner et al. first quantitatively measured lactyl-CoA in hepatoma cells. They showed that lactyl-CoA is quantifiable at 1.14 × 10^-8^ pmol per cell in HepG2 cell culture and 0.0172 pmol mg^-1^ tissue wet weight in mouse heart. These leves are similar to crotonyl-CoA, but significantly less 20-350 times less than majo acyl-CoAs including acetyl-, propionyl- and succinyl-CoA (114). Pan et al. isolated liver cancer stem cells (LCSCs) from MHCCLM3 and Hep3B cell lines with significantly higher lactate levels than those in HCC cells. They identified that the increase of two histone H3 lactylation sites (H3K9la, H3K56la) effectively promoted the progression of HCC. The levels of Pan Kla and histones H3K9la and H3K56la in liver cancer were positively correlated with the expression of cancer malignancy markers (the stemness marker CD133, the proliferation marker BCL2, the cancer cell proliferation marker Ki67, and the glycolysis enzyme LDHA). Inhibition of LDHA was able to reduce lactate levels in LCSCs and inhibit lactylation. Demethylzeylasteral (DML) inhibits LCSC-induced tumorigenicity by inhibiting histone H3 lactylation (112). Intracellular lactate production and histone lactylation levels are elevated under hypoxic conditions, inhibition of pyruvate dehydrogenase and lactate dehydrogenase activities using sodium chloroacetate and sodium oxalate, respectively, could attenuate the hypoxia-induced elevation effect. Under positive oxygen conditions, human lactate production and histone lactylation were completely inhibited in HepG2 cells after knockdown of LDHA and LDHB (9).

### Non-histone lactylation

During the dynamic metabolic homeostasis of tissues, part of the lactate produced by the cells is involved in metabolism, while the other part is received to participate in epigenetic modification and non-histone lactylation. Lactylation was originally discovered on human histones (9), and recent studies have shown that lactylation is a gross modification of human cells and tissues. Digging of the Meltome Atlas revealed that glycolytic enzymes in human cells are heavily lactated, particularly K147 of fructose-bisphosphate aldolase A (ALDOA) (7). Yang et al. identified 2375 Kla sites in 1014 proteins in gastric cancer AGS cells (8). Gu et al. established a solid tumor model of liver cancer by subcutaneously injecting Hepa1-6 cells into B6 mice, and injected lactate dehydrogenase inhibitor (LDHi) to reduce LDH activity. It was found that the lactate concentration in the tumor of the mice was significantly reduced, and the immunomodulatory effect of Treg cells in the TME was inhibited. Lactate levels are elevated, and lactate levels are elevated in Treg cells. Lactate enhances TGF-βR1-mediated TGF-β signaling by regulating the lactylation of Lys72 residues in MOESIN, which is involved in the metabolic reprogramming of Treg cells (111).

Abnormal lactate metabolism is an important feature of liver cancer, and NMR analysis showed that HCC displayed high levels of lactate and low levels of glucose compared with distant non-tumor tissues (NTT) (115). The elevated lactate concentration detected in the serum of HCC patients also confirmed that this is the result of abnormal lactate metabolism in liver cance (116). Taken together, this abnormal lactate metabolism is critical for the maintenance of tumor growth and progression in HCC, and plays an important role in the lactylation of tumor cells histones as well as non-histone proteins.

## Lactate promotes immunosuppressive TME

**Figure 4:**
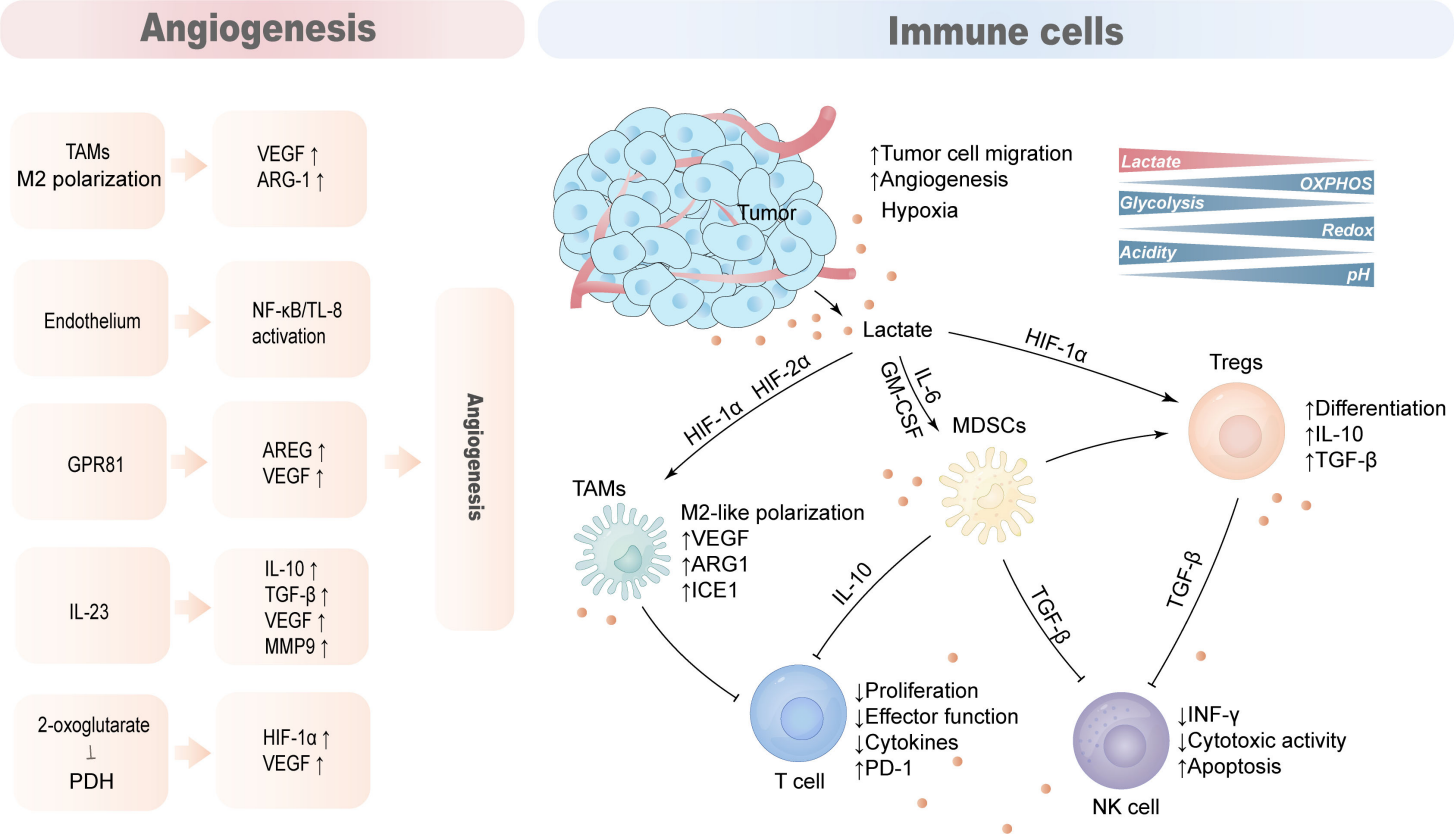
Lactate promotes the production of an immunosuppressive microenvironment. The tumor microenvironment is mainly composed of tumor cells, anti-tumor immune cells, tumor-promoting immune cells, blood vessels and cytokines. Lactate acts as an immunosuppressive factor that hinders cytotoxic action in T cells and NK cells while supporting the immunosuppressive function of TAMs, MDSCs and Tregs to promote tumor immune evasion. Lactate also promotes hypoxia and angiogenesis that aggravates the immunosuppressive nature of TME.

The TME of HCC is composed of complex components such as tumor cells, immune cells, stromal cells, and blood vessels. Due to the Warburg effect, tumor cells secrete lactate into the TME, reducing the pH of the TME. Lactate acts as an immunosuppressive factor to promote tumor progression by hindering T cell and natural killer (NK) cell function or supporting the suppression of TAMs, MDSCs, and regulatory T cells (Tregs) (**Figure 4**).

### T cell

The rate of lactate production and secretion by tumor cells and activated T lymphocytes was significantly increased, and the large amount of lactate and increased acidity of the TME inhibited the proliferation of activated T cells and the production of cytokines (117). Lactate secreted by tumor cells hinders T cell function, especially the lytic function of CD8 T cells, by modulating the redox state of nicotinamide adenine dinucleotide (NAD) and (NADH) (118). Lactate inhibits the proliferation and cytokine production of 95% of human cytotoxic T lymphocytes (CTLs) and reduces cytotoxicity by 50%. A high lactate environment in the TME blocks monocarboxylate transporter-1 (MCT-1) export resulting in abnormal CTL lactate metabolism and impaired function (117).

### NK cell

High levels of lactate in the TME interfere with the secretion of the antitumor cytokines INF-γ, perforin, and granzyme B in T cells and NK cells, thereby promoting tumor immune escape and growth. Lactate-pretreated NK cells inhibited NK cell cytotoxicity by downregulating the expression of NKp46 (119, 120). In addition, high levels of acidic lactate environments are not conducive to the proliferation of natural killer T cells (NKTs) and affect their activity and function (121).

### TAMs

Macrophages regulate immune responses to pathogens, maintain tissue homeostasis and participate in tissue repair and remodeling (122). M1-type macrophages tend to be more pro-inflammatory phenotypes, whereas M2-type macrophages primarily play a role in immune regulation, tissue remodeling, and tumor progression (123). Tumor-associated macrophages (TAMs) typically exhibit pro-inflammatory and anti-tumor activities, and gradually polarize to the M2 phenotype as tumors progress. MCTs take up tumor-derived lactate on the cell membrane of TAMs to induce vascular endothelial growth factor (VEGF), L-arginine arginase-1(ARG1) and the expression of the transcriptional repressor ICER through HIF-1α, and promoting M2-like polarization of TAMs. This process can support tumor growth and suppress antitumor immune responses (124, 125).

### MDSCs

MDSCs are the most prominent myeloid-derived cell population that exerts extensive immunosuppressive functions, inhibiting innate immunity and adaptive immunity in the TME by preventing dendritic cell maturation, inhibiting NK cell toxicity and T cell activation, and promoting Tregs differentiation (126). The number of myeloid-derived suppressor cells (MDSC) is reduced in LDHA knockdown mice, and exogenous lactate increases MDSCs production mediated by GM-CSF and IL-6, and these cells have significant NK inhibitory activity (120). Lactate increases MDSCs activity through GPR81/mTOR/HIF-1α/STAT3 pathway and its inhibition of NK cell, antitumor T cell activity (120, 127).

### Tregs

Regulatory T cells (Tregs) are significantly enriched within tumors, and tumor-infiltrating Tregs require lactate uptake to support their proliferative and immunosuppressive functions (128). Lactate-activated Tregs have reduced glucose uptake, and instead show increased MCT1 to accelerate lactate uptake, increased LDHA activity, and enhanced immunosuppressive capacity (129).

## Lactate mediates the expression of immunosuppressive molecules and their receptors

**Figure 5:**
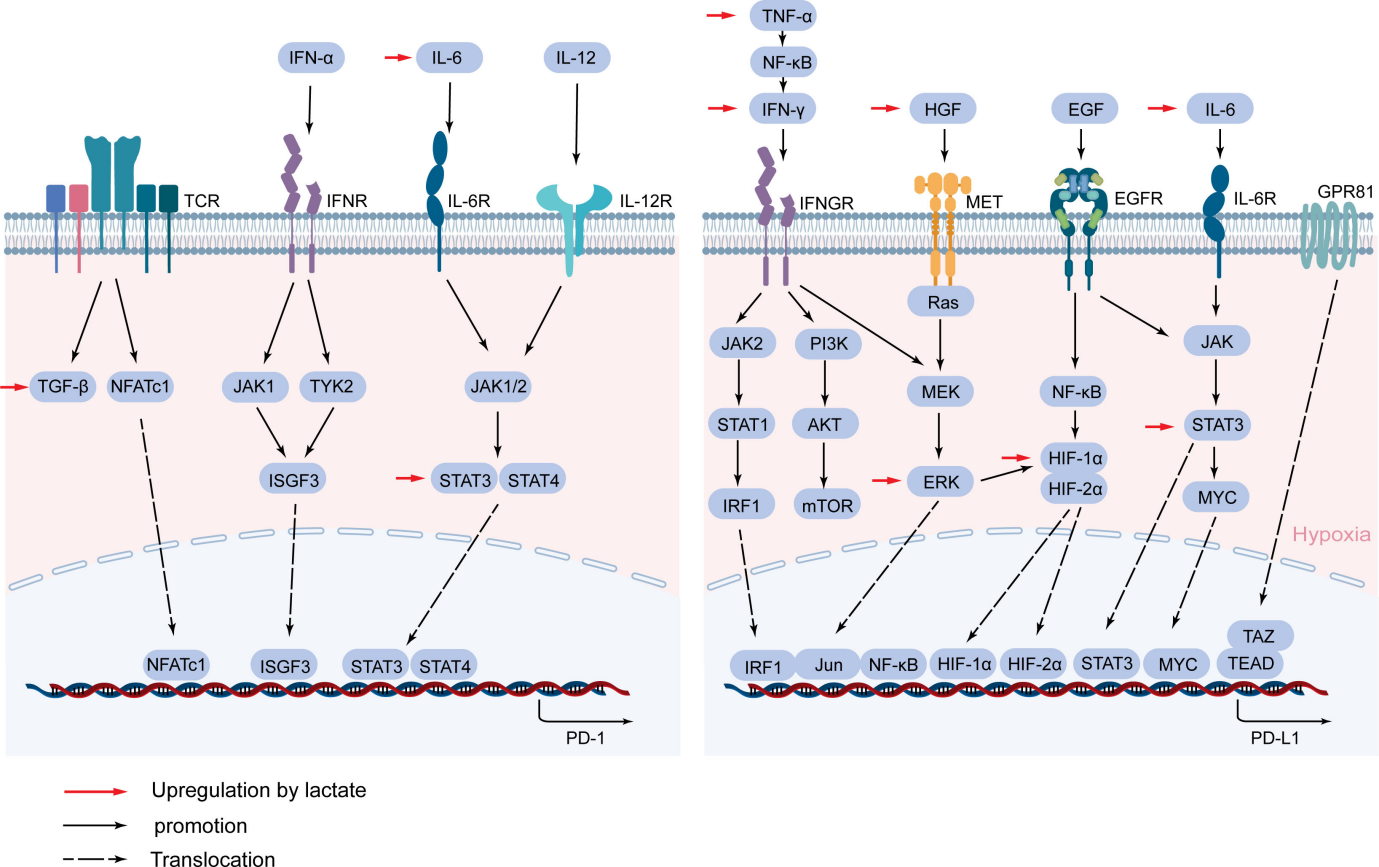
Lactate mediates the expression of PD-1 and PD-L1. PD-1 and PD-L1 are mainly activated by a cascade of specific cytokines and related signaling pathways. Lactate regulates PD-1 and PD-L1 expression through TGF-β/SMAD, IL-6/STAT3, HGF/MET signaling pathways, and cytokines and proteins such as IFN-γ, TNF-α, HIF-1α, and GPR81.

PD-1, as a surface molecule that transmits immunosuppressive signals, is expressed on the surface of immune cells such as activated T cells, B cells, and NK cells (130). Activated PD-1 promotes tumor immune escape by inhibiting the activation of immune cells and the secretion of related antitumor factors (131). PD-1 expression is complexly regulated, and its expression is rapidly induced after T cell receptor (TCR) activation (132). The TGF-β/Smad pathway plays an important role in this process, and blocking TGF-β can significantly inhibit the expression of PD-1 (133). NFATc1 is activated after TCR activation, then NFATc1 enters the nucleus and binds to DHS-C region within the conserved region C (CR-C) located at the transcription initiation point 5’, thereby activating Pdcd1 transcription (134, 135). Blimp-1 inhibits the expression of NFATc1 and displaces it from CR-C, thereby removing the induction effect after TCR activation, resulting in inhibition of PD-1 gene transcription (136). TCR can also promote the expression of PD-1 and attenuate the T cell response in conjunction with IFN-α (137). In macrophages and T cells, IFN-α can promote PD-1 expression through the JAK/STAT signaling pathway (138). IFN-α increases PD-1 expression by activating JAK1 and TYK2 and inducing the binding of ISGF3 complexes (STAT1/STAT2/IRF9) to ISRE located at the promoter CR-C (139). In addition, IL-6 or IL-12 enhances PD-1 expression by changing the structure of chromatin and activating STAT3 and STAT4. Other cytokines in the tumor microenvironment are also able to regulate PD-1 expression, such as gamma-chain cytokines IL-2, IL-7, IL-15, and IL-21. Although these cytokines-induced PD-1 expression does not affect the expansion and survival of peripheral T cells, it can inhibit cytokine secretion in T lymphocytes when TCR is involved (140) (**Figure 5**).

PD-L1 on tumor cells binds to PD-1 on immune cells and mediates negative feedback for various lymphocyte activation (141). In TME, to avoid T cell attack, cancer cells hijack inflammatory factors IFN-γ, TNF-α, IL-6-mediated inflammatory pathways to enhance PD-L1 expression (142–144). IFN-γ is often considered an inducer of PD-L1 and is produced in large quantities when T cells and NK cells are activated (145). IFN-γ binds to its receptor to activate the JAK/STAT pathway, preferentially inducing the expression of the transcription factor interferon response factor 1 (IRF1) *via* STAT1 (146). IRE1/2 constitutes the binding site of IRF1 in the PD-L1 promoter and regulates the transcription of PD-L1 in HCC (147). In addition, the PI3K/AKT pathway, activated by IFN-γ, also upregulated the expression of PD-L1 in tumors (148). IFN-γ can also activate the NF-κB pathway, which in turn mediates the expression of PD-L1 (149). TNF-α can be released by activated TAMs, which is a major driver of inflammation, while it also drives EMTs to regulate PD-L1 expression (150). In addition to EMT, TNF-α upregulates PD-L1 expression by activating NF-κB and ERK1/2 signals (143). The inflammatory factor IL-6 triggers the expression of PD-L1 in the JAK/STAT3 and MEK/ERK signaling pathways (144, 151). Previous studies have also demonstrated that increased IL-6 in HCC activates the STAT3/c-MYC/miR-25-3p pathway, resulting in a decrease in protein tyrosine phosphatase receptor O (PTPRO), which downregulates PD-L1 expression by regulating JAK2-STAT1/3 activation (152). In addition, STAT3 can act directly on the promoter of PD-L1 to regulate the expression of PD-L1 (153).

In addition to the inflammatory signaling pathway, RAS/MEK/ERK signaling can regulate the expression of the PD-L1 gene through crosstalk with inflammatory signaling (132). When MEK is inhibited, IFN-γ-induced STAT1 phosphorylation and PD-L1 transcription are also inhibited (154). Conversely, activation of the MEK/ERK pathway increases PD-L1 expression (132). Hepatocyte growth factor (HGF) activates Met and epidermal growth factor receptor (EGFR) activation also regulate PD-L1 expression through the RAS/MEK/ERK pathway (154–156).

Studies have shown that certain oncogenic signaling pathways can also promote tumor immune escape by driving the expression of PD-L1. Chromatin Immunoprecipitation (ChIP) analysis showed that the oncogene MYC bound to the PD-L1 promoter and directly regulated the expression of PD-L1 at the transcription level (157), and Kim et al. also confirmed that positive expression of MYC correlated with PD-L1 expression in NSCLC (158). The pharmacological inactivation of MYC weakens mRNA levels of PD-L1 and re-establishes anti-tumor immunity in TME (159). Hypoxia is typical of most tumor microenvironments and is achieved by activating a range of hypoxia-inducible factors (HIFs), and this response can also lead to increased expression of PD-L1 (160). Specifically, the promoter of PD-L1 contains HIF-1α response elements, and HIF-1α and HIF-2α have been shown to interact with the hypoxia response element (HRE) in the PD-1 promoter (161, 162), and it have been demonstrated in a variety of tumors (163, 164). NF-κB can induce HIF-1α by directly binding to the promoter of HIF-1α, so the NF-κB pathway can enhance the expression of PD-L1 in synergy with HIF-1α (156, 165). In addition, the interaction of TAZ with the transcription factor TEAD directly regulates the transcription of PD-L1 (166) (**Figure 5**).

## Lactate regulates the expression of PD-1 and PD-1

In TME with high glycolysis in hepatocellular carcinoma, lactate inhibits the function of effector T cells as well as activates the function of immunosuppressive cells (e.g., Treg) by directly upregulating the expression of PD-1 and PD-L1 (167). In addition to direct regulation, the expression of PD-1 and PD-L1 is mainly activated by the cascade of the above cytokines and related signaling pathways, while lactate has been shown to be involved in multiple signaling pathways that can activate PD-1 and PD-L1 expression(**Figure 5**).

### TGF-β/SMAD pathway

TGF-β is involved in regulating PD-1 transcription, its expression increases in cancer in a lactate-dependent manner, accumulates in the tumor microenvironment, and TGF-β function is also increased by the increase of lactate (168, 169). Lactate upregulates the TGF-β/Smad pathway and coordinates the expression of TGF-β1/Snail and TZ/AP-1 to activate EMT-related genes (170, 171). Lactate-induced oxidoreductase NOX2 mediates TGF-β activation, promotes phosphorylation of TGF-β receptors and subsequent Smad 2/3-Smad4 colocalization (172). TGF-β attenuates tumor response to PD-L1 blockade by promoting the exclusion of T cells. In mice with progressive liver metastases, blocking TGF-β signaling increased tumor sensitivity against PD-1/PD-L1 therapy, promoting T cell penetration into tumor centers to function (173, 174). In addition, lactate regulates TGF-β and downstream SMAD3 signaling in regulatory T cells through MOESIN lactylation (111). In summary, there may be a regulatory cascade between lactate and PD-1 or PD-L1, with TGF-β functioning as an intermediate molecule. As a positive feedback loop that promotes lactate-TGF-β signaling cycling as a danger signal, tumor cells may produce more TGF-β promote PD-1 expression, thus evading immune surveillance (168).

### IL-6/STAT3

High concentrations of IL-6 in the tumor microenvironment were identified as one of the main causes of cancer growth, and lactate plays an important role in the expression of IL-6 and the activation of the STAT3 signaling pathway. Extensive studies have shown that lactate-induced acidosis promotes IL-6 expression, possibly due to acidosis activating ERK1/2 and p38 signaling in cells (175, 176). Lactate mediates partial crosstalk between tumor cells and macrophages, and also promotes the secretion of IL-6 and upregulates the expression of HIF1α (177). Higher IL-6 levels were also detected in patients with high expression of H3K18la, indicating that lactate and lacttylation modifications jointly regulate IL-6 secretion (102). In addition, a significant correlation was observed between IL-6 and lactate dehydrogenase (178). Lactate-induced IL-6/STAT3 signaling in inflammatory macrophages occurs simultaneously with histone lactylation (93), and when the IL-6/STAT3 pathway is inhibited, lactate production is reduced in turn (179).

Lactate also activates the STAT3 signaling pathway independently of IL-6, and lactate enhances STAT3 expression through ERK1/2 as well as phosphorylation of EZH2 enhancers (180, 181). Lactate not only enhances mRNA levels of TGF-β, but also promotes M2 macrophage polarization by accelerating p-STAT3, while STAT3 inhibitors eliminate this lactate salt-mediated macrophage polarization (169). Whole-cell lysates that block STAT3 stimulate the activation of T cells and NK cells and enhance the infiltration of toxic CD8 T cells in HCC tumor tissue, also reducing TGF-β production (182).

### HGF/MET

HGF and its receptor MET play a key role in the occurrence and metastasis of liver cancer, and lactate can regulate the expression of HGF (183). Lactate produced by tumors leads to an increase in HGF in NF-κB in cancer-associated fibroblasts, which in turn activates MET/Ras/REK signaling in tumors (184, 185). MCT1 regulates lactate transport and knocks down the expression of MCT1, resulting in blockage of signaling of HGF receptor MET. How lactate regulates the expression of PD-L1 through HGF/MET deserves further study (186).

### IFN-γ

IFN-γ appears to play a dual role in the tumor microenvironment, synergistic with granzyme B-mediating tumor killing of toxic T cells (187). On the other hand, IFN-γ also mediates the expression of immunosuppressive molecules to promote tumor immune escape, and lactate participates in regulating this process (145). In HCC, tissues with high IFN-γ characteristics are often accompanied by elevated expression of PD-L1 (145). Lactate accumulation at the site of chronic inflammation not only directly promotes PD-1 expression, but may also upregulate PD-1 by promoting IFN-γ transcription (188). In tumors, lactate significantly upregulated IFN-γ levels of M2 tumor-associated macrophages and promoted apoptosis of T cells through the PD-1/PD-L1 pathway (189). LDHA is a rate-limiting enzyme for lactate production processes, and LDHA promotes IFN-γ expression through histone acetylation in epigenetic modifications (190).

### TNF-α

In solid tumors, lactate accumulation leads to acidification of the tumor microenvironment, affecting the function and phenotype of cells in the microenvironment (177). Among them, lactate-mediated acidic environment significantly upregulates the expression of TNF-α, an inflammatory mediator, and activates ERK1/2 signaling (175). HCC tumor tissues with high glycolytic macrophages showed higher glycolysis rates, produced more lactate, and mediated the upregulation of PD-L1 induced by inflammatory factors such as TNF-α, blocking TNF-α which could inhibit the expression of PD-L1 in 40%-50% of tumor macrophages (191).

### HIF-1α

In HCC tumor tissues, PD-L1 is produced in a HIF-1α-dependent manner by macrophages with a high glycolytic phenotype (191). Even if tumor cells metabolize glucose through the “Warburg effect”, the accumulation of its product lactate salts will further induce hypoxia, which in turn will further promote lactate production, and HIF-1α is an important regulator of this process (192). The expression of HIF-1α protein increased significantly in THP-1 macrophages co-cultured with cancer cells treated with lactate, and the HIF-1α pathway was involved in coordinating PD-L1-mediated immune escape. After transfection of THP-1 cells with HIF-1α siRNA, the redistribution of M2-TAM subsets and the expression of PD-L1 were reversed (189). HIF-1α is essential for lactate-mediated activation of GPR81/mTOR/HIF-1α/STAT3 pathway, and inhibition of lactate production in tumor cells or HIF-1α expression in MDSC can restore the immune response of antitumor T cells (127). When the immunosuppressive factor macrophage migration inhibitor (MIF) is inhibited, the lactate production of melanoma cells is significantly reduced, and the expression of HIF-1α and PD-L1 is also significantly reduced (193).

### GPR81

Lactate evades the surveillance of the immune system by activating GPR81 in tumor cells to induce the production of PD-L1 in tumor cells (194). Lactate-mediated activation of GPR81 reduces intracellular cAMP levels and inhibits protein kinase A (PKA) activity, leading to activation of the transcriptional coactivator TAZ, while TAZ/YAP/TEAD enhances PD-L1 promoter activity (195). The double blockade of lactate/GPR81 and PD-1/PD-L1 significantly increased the antitumor effect of metformin and even caused tumor regression (196). In addition, lactate-activated STAT3 is also able to directly activate the GPR81 promoter and activate its expression (181). This also proves that as a powerful transcription factor, lactate-mediated STAT3 can not only directly induce the expression of PD-1 and PD-L1, but also activate the expression of PD-1 and PD-L1 by other regulatory genes.

## Lactate promotes hypoxia and angiogenesis

In the hypoxic tumor microenvironment, hypoxia-inducible factor 1 (HIF1) promotes hypoxic glycolysis and angiogenesis by binding to its receptor, which in turn further aggravates hypoxia. Lactate released by tumor cells activates angiogenesis-promoting signaling and is a well-established promoter of angiogenesis. Overall, lactate is involved in angiogenesis through the following mechanisms (1): Induced polarization of TAMs to M2 phenotype, increased expression of VEGF and Arg1, and thus stimulated angiogenesis (197) (2); MCT1-mediated activation of the NF-κB/IL-8 pathway in endothelial cells drives endothelial cells to form blood vessels (198) (3); Activation of GPR81 increases the secretion of AREG, which further increases the production of VEGF and promotes angiogenesis (79) (4); Stimulate the production of cytokine IL-23, which further induces the expression of IL-10, TGF-β, VEGF and MMP9 (199, 200) (5); Support the activation of HIF-1α and upregulate VEGF by inhibiting prolyl hydroxylase (PHD) through 2-oxoglutarate (192, 201).

## The future of anti-lactate combined with immunotherapy for HCC

The efficacy and safety of immunotherapy in the treatment of solid tumors make it an ideal treatment option for the treatment of HCC. So far, a variety of immunotherapies have been clinically tested and achieved effective results, such as immune checkpoint inhibitors (ICIs), which have become mature HCC treatments (202).

**Figure 6:**
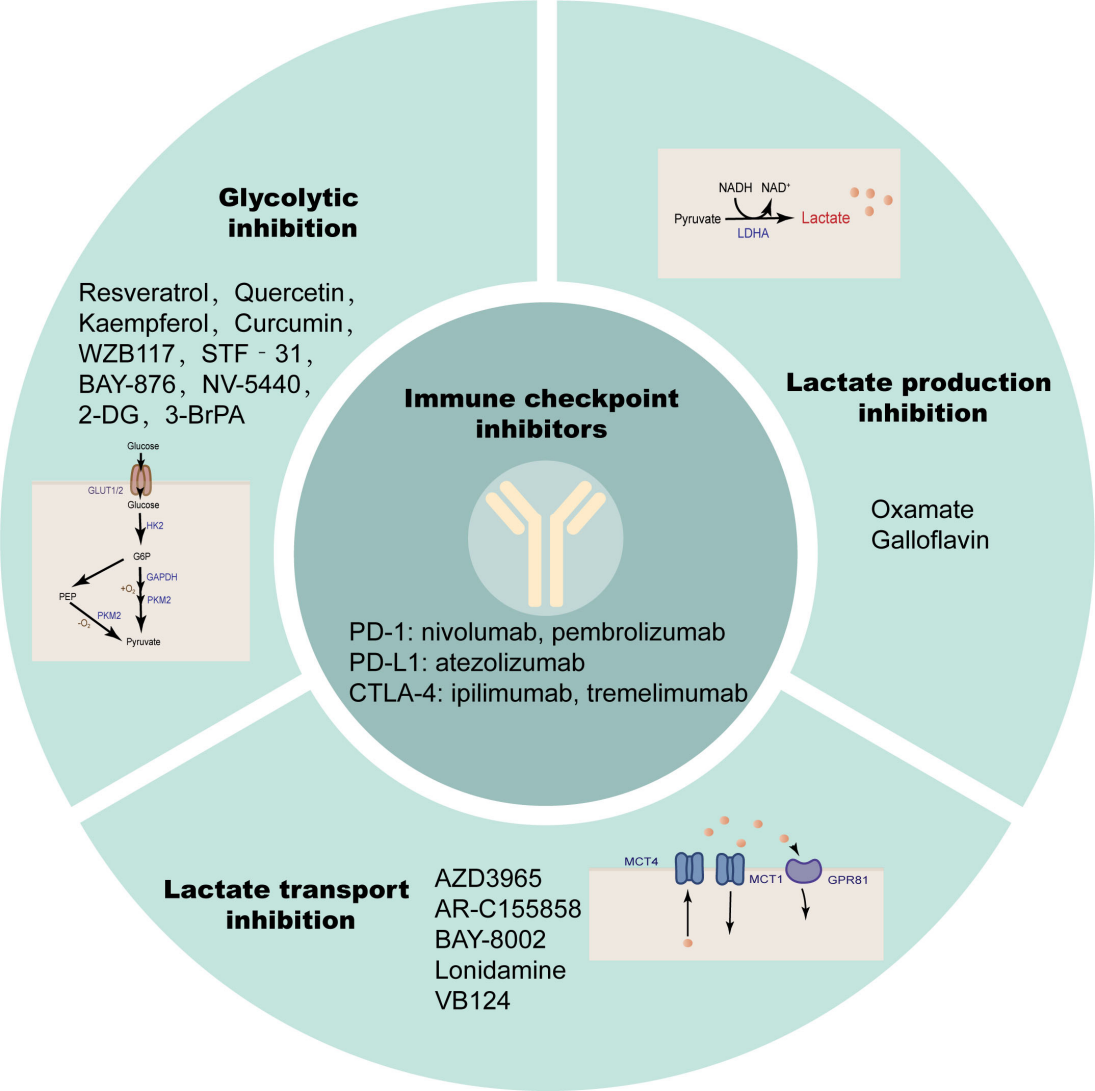
Anti-lactate therapy combined with ICIs. Single immune checkpoint inhibitors are less effective in treatment, while simultaneous targeting of glycolysis, lactate production, and transport are more effective.

Immune checkpoints are surface molecules that transmit inhibitory signals on the surface of immune cells, including but not limited to programmed cell death protein-1 (PD-1), cytotoxic T lymphocyte antigen 4 (CTLA-4), T cell Ig and ITIM domain (TIGIT), T cell immunoglobulin domain and mucin domain-3 (TIM-3), Lymphocyte activation gene 3 (LAG3), B and T lymphocyte attenuator (BTLA) (203). Solid tumors, including HCC, evade antitumor immune responses through such inhibitory immune receptors (204). PD-1 and CTLA-4 are members of the CD28 family, expressed on most immune cells, and by binding to their ligands, transmit inhibitory signals to T cells to promote tumor immune escape (205, 206). ICIs are monoclonal antibodies that can block the binding of immune checkpoints to their ligands and block the transduction of inhibitory signals, thereby restoring the activity of T cells to exert immune recognition and immune attack to enhance anti-tumor immune responses. The PD-1 inhibitors nivolumab (207) and pembrolizumab (208), PD-L1 inhibitor atezolizumab (209), and the CTLA-4 inhibitors ipilimumab (210) and tremelimumab (211) have been tested individually or in combination in large clinical trials. The results suggest that some patients have lower response rates to ICI therapy alone due to a lack of tumor-infiltrating T cells. The immunotherapy effect of single ICIs has not been satisfactory, and the above findings suggest that lactate modulates the immune response in the TME by modulating the pH of the TME, lactate-dependent pathways, lactate-mediated signaling, and histone modifications. Therefore, anti-lactate combined with immunotherapy has broad prospects (**Figure 6**; **Table 2**).

**Table 2:** Some of the anti-lactate drugs that have been reported.

Type	Inhibitors	Targets	Reference
glycolysis	2-DG	HK2	(212, 213)
glycolysis	3-BrPA	HK2	(214–216)
lactate production	Oxamate	LDHA	(217)
lactate production	Quinoline-3-sulfonamides	LDHA/LDHB	(218)
lactate production	Galloflavin	LDHA	(219)
lactate transport	AZD3965	MCT1	(220)
lactate transport	AR-C155858	MCT1	(221)
lactate transport	BAY-8002	MCT1	(222)
lactate transport	Lonidamine	MCT1/4	(223–225)
lactate transport	VB124	MCT1	(226)

## Targeting glycolysis

Active glycolysis is an important source of lactate, and inhibition of glycolysis appears to be more capable of suppressing lactate due to the “Warburg effect” in cancer cells. Glucose uptake is the first rate-limiting step in glycolysis, and inhibition of glucose transporters may have therapeutic benefits in the treatment of hyperglycolytic tumors. Small-molecule GLUT1 inhibitors such as the natural products Resveratrol (227, 228), Quercetin (229, 230), Kaempferol (231) and Curcumin (232), and the non-natural products WZB117 (233, 234), STF‐31 (235, 236), BAY-876 (237), NV-5440 (238) can effectively inhibit the progression of various cancers including HCC. 2-Deoxy-D-glucose (2-DG) as a glucose analog is a competitive inhibitor of glucose metabolism (239). Studies have shown that 2-DG and sorafenib synergistically inhibit the proliferation of HCC cells and sorafenib resistance (212, 213). 3-Bromopyruvate(3-BrPA), a HK2 inhibitor, reduces the proliferation and motility of HCC cells. It is also able to enhance the efficacy of sorafenib in an *in vivo* model of HCC and can also be considered as a potential clinical chemosensitizer to optimize the index of CNU treatment (214–216). These glycolysis inhibitors also require extensive clinical trials to evaluate their safety and efficacy in patients.

## Target lactate synthesis

Lactate accumulation mediated by abnormally high expression of LDHA is a common and major feature of cancer metabolism (240), so targeting LDHA is a safe and effective strategy that has been tested clinically. Oxamate as an inhibitor of LDHA enhanced the antitumor activity of sorafenib, imatinib and sunitinib in HCC (217). Quinoline-3-sulfonamides and Galloflavin, which are inhibitors of LDHA, can inhibit HCC tumor proliferation (218, 219). LDH levels can also predict the prognosis of patients with a variety of ICI-treated tumors, such as patients with advanced esophageal squamous cell carcinoma (ESCC) and non-small cell lung cancer (241, 242).

## Target lactate transport

MCT1/4 and GPR81 mediate the exchange of lactate on the tumor cell membrane and are also important factors in tumor aggressiveness, and blocking lactate transport is a potential target for cancer therapy (79, 243). Dual blockade of the lactate/GPR81 pathway and the PD-1/PD-L1 pathway significantly inhibits tumor growth and induces tumor regression, while increasing the number of CD8 T cells in tumor tissue and increasing IFN-γ secretion in lymph nodes (196). In lactate-mediated TME, PD-1 is more expressed in Tregs than in effector T cells. MCT1 upregulation in Tregs and its downstream lactate signaling promote resistance to anti-PD-1 therapy in HCC patients (244). MCT1 inhibitor AZD3965 was combined with anti-ICIs to enhance the immunity of antigen-specific CD8 T cells to tumors, effectively inhibiting tumor growth (220). AR-C155858, BAY-8002 and Londonamine (LND) also showed effective MCT1 inhibitory and immunomodulatory activities, and the cytotoxicity of some anti-tumor drugs (such as anti-PD-1) of HCC was increased after LND treatment (221–225). MCT4 targeted inhibition destroys intracellular pH homeostasis and initiates self-apoptosis of HCC cells (245), and MCT4 inhibitor VB124 enhances T cell infiltration and the potency of anti-PD-1 immunotherapy in HCC mice (226).

In addition to targeting glycolysis, lactate production and transport, targeting mTOR pathways also play an important role in regulating lactate metabolism. Water-soluble rapamycin analogues (temsirolimus, everolimus), ATP-competitive mTOR inhibitors (MLN0128, AZD2014, PP242), and dual PI3K/mTOR inhibitors (NVP-BEZ235, LY3023414, PQR309, XH00230381967, SN20229799306, GSK2126458, PKI-587) have been used to treat a variety of cancers (246). Although the efficacy of mTOR inhibitors alone is limited, mTOR inhibitors exert anti-HCC tumor activity in synergy with anti-PD-1 therapy (247). The concentration of lactate in the tumors of HCC individuals who responded to PD-1 inhibitor therapy and the level of MOESIN lactylation in Treg cells were lower than in the unresponsive individuals. Anti-lactate combined with immunotherapy has a stronger anti-tumor effect, so ICIs and anti-lactate combination therapy is a potential treatment direction (111).

## Conclusion

Multiple evidence shows that lactate plays an important role in regulating tumor cell metabolic reprogramming, remodeling TME, and regulating anti-tumor immunity, and lactate modification is an important way for lactate to function. Metabolic reprogramming resulting in active glycolysis increases lactate levels and lactylation in the TME. The production and accumulation of lactate plays a key role in HCC. We believe that accelerated uptake of glucose and aerobic glycolysis in tumor tissue increases the production, accumulation and release of lactate. Acidification of the TME and persistently high lactate levels lead to abnormal signaling that promotes the formation of an immunosuppressive tumor microenvironment, thereby supporting tumor immune escape. One of the consequences of abnormal lactate metabolism is an abnormal increase in lactylation levels, and the increase in lactylation levels can be observed in a variety of diseases and cancers.

Cancer immunotherapy seems to be one of the most promising treatment modalities over the past decade. At present, immune checkpoint PD-1/PD-L1 inhibitors have been studied in multiple clinical trials, but only a few patients have benefited from them. Lactate not only plays a role in TME as an immunosuppressive molecule, but also participates in regulating the expression of other immunosuppressive molecules such as PD-1 and PD-L1, thereby exerting immunosuppressive effects and affecting the therapeutic effect of immune checkpoint inhibitors. Therefore anti-lactate combined immunotherapy appears to be a more promising treatment modality. In addition to targeting glycolysis, lactate synthesis and transport, epigenetic modifying enzymes may also be new therapeutic targets. Overall, targeting lactate metabolic pathways combined with immune checkpoint inhibitors may be able to more selectively modulate immune cell activity, and lactate modification may be beneficial for in-depth study of more diseases and their processes.

These findings about lactylation are still in their infancy, only lactylation is known to be written into enzyme p300, it is not clear which enzymes recognize lactylation, and more lactylation “writers” as well as “readers” and “erasers” have yet to be discovered. Studies have shown that in a variety of cancers, key enzymes involved in glucose metabolism are rich in non-histone lactate salt modifications, how do they in turn regulate lactate metabolism? Is there crosstalk between lactate modifications and other PTMs? These details all deserve further study.

## Author contributions

YX collected the related papers and drafted the manuscript. XH, YR, QX, and XL participated in the design of the review. SS and YW initiated the study and revised and finalized the manuscript. All authors contributed to the article and approved the submitted version.

## Glossary

**Table:**

PTM	Post-translational modification
HCC	Hepatocellular carcinom
TME	Tumor microenvironment
MAFLD	Metabolic associated fatty liver
NAFLD	Nonalcoholic fatty liver disease
NASH	Nonalcoholic steatohepatitis
CO2	Carbon dioxide
TCA	Tricarboxylic acid
PEP	Phosphoenolpyruvate
LDHA	Lactate dehydrogenase A
GDH	Glutamate dehydrogenase
DC	Dendritic cells
MCT	Monocarboxylate transporter
HK	Hexokinase
GAPDH	Glyceraldehyde-3-phosphate dehydrogenase
PKM2	Pyruvate kinase 2
LDH	Lactate dehydrogenase
TACE	Transarterial-chemoembolization
GPR81	G protein-coupled receptor 81
PDK	Pyruvate dehydrokinase
PDH	Pyruvate dehydrogenase
HIF-1α	Hypoxia-inducible factor 1α
PCAF	P300/CBP-associated factor
NK	Natural killer
Tregs	Regulatory T cells
CTLs	Cytotoxic T lymphocytes (CTLs)
NKTs	Natural killer T cells
TAMs	Tumor-associated macrophages
VEGF	Vascular endothelial growth factor
ARG1	Arginase-1
MDSCs	Myeloid-derived suppressor cells
PHD	Prolyl hydroxylase
BMDM	Bone marrow-derived macrophages
INF-γ	Interferon gamma
LGSH	Lactyl-glutathione
GLO2	Glyoxalase 2
HDAC	Class I histone deacetylases
BCAP	Phosphoinositide 3-kinase
TIMs	Tumor-infiltrating myeloid cells
NSCLC	Non-small cell lung cancer
VHL	Inactive von Hippel-Lindau
ccRCC	Clear cell renal cell carcinoma
PDGFRβ	Platelet-derived growth factor receptor beta
LCSCs	Liver cancer stem cells
ALDOA	Aldolase A
LDHi	Lactate dehydrogenase inhibitor
NTT	Non-tumor tissues
PD-1	Programmed cell death protein-1
CTLA-4	Cytotoxic T lymphocyte antigen
TIGIT	T cell Ig and ITIM domain
TIM-3	T cell immunoglobulin domain and mucin domain-3
LAG3	Lymphocyte activation gene 3
BTLA	B and T lymphocyte attenuator
2-DG	2-Deoxy-D-glucose
3-BrPA	3-Bromopyruvate
ESCC	Esophageal squamous cell carcinoma

## References

[1] Ferguson BS Rogatzki MJ Goodwin ML Kane DA Rightmire Z Gladden LB. Lactate metabolism: historical context, prior misinterpretations, and current understanding. Eur J Appl Physiol (2018) 118(4):691–728. doi: 10.1007/s00421-017-3795-6 29322250

[2] Li X Yang Y Zhang B Lin X Fu X An Y. Lactate metabolism in human health and disease. Signal Transduct Target Ther (2022) 7(1):305. doi: 10.1038/s41392-021-00847-2 36050306 PMC9434547

[3] Certo M Tsai CH Pucino V Ho PC Mauro C. Lactate modulation of immune responses in inflammatory versus tumour microenvironments. Nat Rev Immunol (2021) 21(3):151–61. doi: 10.1038/s41577-020-0406-2 32839570

[4] Warburg O Wind F Negelein E. The metabolism of tumors in the body. J Gen Physiol (1927) 8(6):519–30. doi: 10.1085/jgp.8.6.519 PMC2140820 19872213

[5] Vander Heiden MG Cantley LC Thompson CB. Understanding the warburg effect: the metabolic requirements of cell proliferation. Science (2009) 324(5930):1029–33. doi: 10.1126/science.1160809 PMC2849637 19460998

[6] Liberti MV Locasale JW. Histone lactylation: A new role for glucose metabolism. Trends Biochem Sci (2020) 45(3):179–82. doi: 10.1016/j.tibs.2019.12.004 31901298

[7] Wan N Wang N Yu S Zhang H Tang S Wang D. Cyclic immonium ion of lactyllysine reveals widespread lactylation in the human proteome. Nat Methods (2022) 19(7):854–64. doi: 10.1038/s41592-022-01523-1 35761067

[8] Yang D Yin J Shan L Yi X Zhang W Ding Y. Identification of lysine-lactylated substrates in gastric cancer cells. iScience (2022) 25(7):104630. doi: 10.1016/j.isci.2022.104630 35800753 PMC9253728

[9] Zhang D Tang Z Huang H Zhou G Cui C Weng Y. Metabolic regulation of gene expression by histone lactylation. Nature (2019) 574(7779):575–80. doi: 10.1038/s41586-019-1678-1 PMC6818755 31645732

[10] Chen Y Ramjiawan RR Reiberger T Ng MR Hato T Huang Y. CXCR4 inhibition in tumor microenvironment facilitates anti-programmed death receptor-1 immunotherapy in sorafenib-treated hepatocellular carcinoma in mice. Hepatology (2015) 61(5):1591–602. doi: 10.1002/hep.27665 PMC4406806 25529917

[11] Dong N Shi X Wang S Gao Y Kuang Z Xie Q. M2 macrophages mediate sorafenib resistance by secreting HGF in a feed-forward manner in hepatocellular carcinoma. Br J Cancer (2019) 121(1):22–33. doi: 10.1038/s41416-019-0482-x 31130723 PMC6738111

[12] Tao Z Ruan H Sun L Kuang D Song Y Wang Q. Targeting the YB-1/PD-L1 axis to enhance chemotherapy and antitumor immunity. Cancer Immunol Res (2019) 7(7):1135–47. doi: 10.1158/2326-6066.CIR-18-0648 31113805

[13] Yao W Ba Q Li X Li H Zhang S Yuan Y. A natural CCR2 antagonist relieves tumor-associated macrophage-mediated immunosuppression to produce a therapeutic effect for liver cancer. EBioMedicine (2017) 22:58–67. doi: 10.1016/j.ebiom.2017.07.014 28754304 PMC5552238

[14] Zhou SL Zhou ZJ Hu ZQ Huang XW Wang Z Chen EB. Tumor-associated neutrophils recruit macrophages and T-regulatory cells to promote progression of hepatocellular carcinoma and resistance to sorafenib. Gastroenterology (2016) 150(7):1646–58 e17. doi: 10.1053/j.gastro.2016.02.040 26924089

[15] Eslam M Sanyal AJ George J International Consensus P. MAFLD: A consensus-driven proposed nomenclature for metabolic associated fatty liver disease. Gastroenterology (2020) 158(7):1999–2014 e1. doi: 10.1053/j.gastro.2019.11.312 32044314

[16] Forner A Reig M Bruix J. Hepatocellular carcinoma. Lancet (2018) 391(10127):1301–14. doi: 10.1016/S0140-6736(18)30010-2 29307467

[17] Jeppesen JB Mortensen C Bendtsen F Moller S. Lactate metabolism in chronic liver disease. Scand J Clin Lab Invest (2013) 73(4):293–9. doi: 10.3109/00365513.2013.773591 23514017

[18] Ha TS Shin TG Jo IJ Hwang SY Chung CR Suh GY. Lactate clearance and mortality in septic patients with hepatic dysfunction. Am J Emerg Med (2016) 34(6):1011–5. doi: 10.1016/j.ajem.2016.02.053 26976769

[19] Shang RZ Qu SB Wang DS. Reprogramming of glucose metabolism in hepatocellular carcinoma: Progress and prospects. World J Gastroenterol (2016) 22(45):9933–43. doi: 10.3748/wjg.v22.i45.9933 PMC5143760 28018100

[20] Hay N. Reprogramming glucose metabolism in cancer: can it be exploited for cancer therapy? Nat Rev Cancer (2016) 16(10):635–49. doi: 10.1038/nrc.2016.77 PMC5516800 27634447

[21] Xia H Huang Z Wang Z Liu S Zhao X You J. Glucometabolic reprogramming: From trigger to therapeutic target in hepatocellular carcinoma. Front Oncol (2022) 12:953668. doi: 10.3389/fonc.2022.953668 35912218 PMC9336635

[22] Liberti MV Locasale JW. The warburg effect: How does it benefit cancer cells? Trends Biochem Sci (2016) 41(3):211–8. doi: 10.1016/j.tibs.2015.12.001 PMC4783224 26778478

[23] DeBerardinis RJ Cheng T. Q's next: the diverse functions of glutamine in metabolism, cell biology and cancer. Oncogene (2010) 29(3):313–24. doi: 10.1038/onc.2009.358 PMC2809806 19881548

[24] DeBerardinis RJ Mancuso A Daikhin E Nissim I Yudkoff M Wehrli S. Beyond aerobic glycolysis: transformed cells can engage in glutamine metabolism that exceeds the requirement for protein and nucleotide synthesis. Proc Natl Acad Sci U S A. (2007) 104(49):19345–50. doi: 10.1073/pnas.0709747104 PMC2148292 18032601

[25] Damiani C Colombo R Gaglio D Mastroianni F Pescini D Westerhoff HV. A metabolic core model elucidates how enhanced utilization of glucose and glutamine, with enhanced glutamine-dependent lactate production, promotes cancer cell growth: The WarburQ effect. PloS Comput Biol (2017) 13(9):e1005758. doi: 10.1371/journal.pcbi.1005758 28957320 PMC5634631

[26] Feron O. Pyruvate into lactate and back: from the warburg effect to symbiotic energy fuel exchange in cancer cells. Radiother Oncol (2009) 92(3):329–33. doi: 10.1016/j.radonc.2009.06.025 19604589

[27] Hensley CT Wasti AT DeBerardinis RJ. Glutamine and cancer: cell biology, physiology, and clinical opportunities. J Clin Invest (2013) 123(9):3678–84. doi: 10.1172/JCI69600 PMC3754270 23999442

[28] Pearce EJ Everts B. Dendritic cell metabolism. Nat Rev Immunol (2015) 15(1):18–29. doi: 10.1038/nri3771 25534620 PMC4495583

[29] Frauwirth KA Riley JL Harris MH Parry RV Rathmell JC Plas DR. The CD28 signaling pathway regulates glucose metabolism. Immunity (2002) 16(6):769–77. doi: 10.1016/S1074-7613(02)00323-0 12121659

[30] Jacobs SR Herman CE Maciver NJ Wofford JA Wieman HL Hammen JJ. Glucose uptake is limiting in T cell activation and requires CD28-mediated akt-dependent and independent pathways. J Immunol (2008) 180(7):4476–86. doi: 10.4049/jimmunol.180.7.4476 PMC2593791 18354169

[31] Tan Z Xie N Banerjee S Cui H Fu M Thannickal VJ. The monocarboxylate transporter 4 is required for glycolytic reprogramming and inflammatory response in macrophages. J Biol Chem (2015) 290(1):46–55. doi: 10.1074/jbc.M114.603589 25406319 PMC4281748

[32] Amann T Maegdefrau U Hartmann A Agaimy A Marienhagen J Weiss TS. GLUT1 expression is increased in hepatocellular carcinoma and promotes tumorigenesis. Am J Pathol (2009) 174(4):1544–52. doi: 10.2353/ajpath.2009.080596 PMC2671384 19286567

[33] Daskalow K Pfander D Weichert W Rohwer N Thelen A Neuhaus P. Distinct temporospatial expression patterns of glycolysis-related proteins in human hepatocellular carcinoma. Histochem Cell Biol (2009) 132(1):21–31. doi: 10.1007/s00418-009-0590-4 19350262

[34] Mathupala SP Rempel A Pedersen PL. Glucose catabolism in cancer cells: identification and characterization of a marked activation response of the type II hexokinase gene to hypoxic conditions. J Biol Chem (2001) 276(46):43407–12. doi: 10.1074/jbc.M108181200 11557773

[35] Yun J Rago C Cheong I Pagliarini R Angenendt P Rajagopalan H. Glucose deprivation contributes to the development of KRAS pathway mutations in tumor cells. Science (2009) 325(5947):1555–9. doi: 10.1126/science.1174229 PMC2820374 19661383

[36] Barthel A Okino ST Liao J Nakatani K Li J Whitlock JP Jr.. Regulation of GLUT1 gene transcription by the serine/threonine kinase Akt1. J Biol Chem (1999) 274(29):20281–6. doi: 10.1074/jbc.274.29.20281 10400647

[37] Gong L Cui Z Chen P Han H Peng J Leng X. Reduced survival of patients with hepatocellular carcinoma expressing hexokinase II. Med Oncol (2012) 29(2):909–14. doi: 10.1007/s12032-011-9841-z 21279699

[38] Zhang Z Huang S Wang H Wu J Chen D Peng B. High expression of hexokinase domain containing 1 is associated with poor prognosis and aggressive phenotype in hepatocarcinoma. Biochem Biophys Res Commun (2016) 474(4):673–9. doi: 10.1016/j.bbrc.2016.05.007 27155152

[39] DeWaal D Nogueira V Terry AR Patra KC Jeon SM Guzman G. Hexokinase-2 depletion inhibits glycolysis and induces oxidative phosphorylation in hepatocellular carcinoma and sensitizes to metformin. Nat Commun (2018) 9(1):446. doi: 10.1038/s41467-017-02733-4 29386513 PMC5792493

[40] Dou C Mo H Chen T Liu J Zeng Y Li S. ZMYND8 promotes the growth and metastasis of hepatocellular carcinoma by promoting HK2-mediated glycolysis. Pathol Res Pract (2021) 219:153345. doi: 10.1016/j.prp.2021.153345 33517164

[41] Li Y Lu Z Liang Z Ji D Zhang P Liu Q. Metastasis-associated in colon cancer-1 is associated with poor prognosis in hepatocellular carcinoma, partly by promoting proliferation through enhanced glucose metabolism. Mol Med Rep (2015) 12(1):426–34. doi: 10.3892/mmr.2015.3416 25738944

[42] Chen W Li Y Zhong J Wen G. Circ-PRKCI targets miR-1294 and miR-186-5p by downregulating FOXK1 expression to suppress glycolysis in hepatocellular carcinoma. Mol Med Rep (2021) 23(6):464. doi: 10.3892/mmr.2021.12103 33880589 PMC8097765

[43] Liao W Liu J Zhang D Huang W Chen R. Butein inhibited *In vitro* hexokinase-2-Mediated tumor glycolysis in hepatocellular carcinoma by blocking epidermal growth factor receptor (EGFR). Med Sci Monit (2018) 24:3283–92. doi: 10.12659/MSM.906528 PMC5987617 29777095

[44] Yu Q Dai W Ji J Wu L Feng J Li J. Sodium butyrate inhibits aerobic glycolysis of hepatocellular carcinoma cells *via* the c-myc/hexokinase 2 pathway. J Cell Mol Med (2022) 26(10):3031–45. doi: 10.1111/jcmm.17322 PMC9097842 35429101

[45] Guo C Liu S Sun MZ. Novel insight into the role of GAPDH playing in tumor. Clin Transl Oncol (2013) 15(3):167–72. doi: 10.1007/s12094-012-0924-x 22911551

[46] Ganapathy-Kanniappan S Kunjithapatham R Geschwind JF. Glyceraldehyde-3-phosphate dehydrogenase: a promising target for molecular therapy in hepatocellular carcinoma. Oncotarget (2012) 3(9):940–53. doi: 10.18632/oncotarget.623 PMC3660062 22964488

[47] Liu S Sun Y Jiang M Li Y Tian Y Xue W. Glyceraldehyde-3-phosphate dehydrogenase promotes liver tumorigenesis by modulating phosphoglycerate dehydrogenase. Hepatology (2017) 66(2):631–45. doi: 10.1002/hep.29202 28387968

[48] Wong CC Au SL Tse AP Xu IM Lai RK Chiu DK. Switching of pyruvate kinase isoform l to M2 promotes metabolic reprogramming in hepatocarcinogenesis. PloS One (2014) 9(12):e115036. doi: 10.1371/journal.pone.0115036 25541689 PMC4277479

[49] Chen Z Lu X Wang Z Jin G Wang Q Chen D. Co-Expression of PKM2 and TRIM35 predicts survival and recurrence in hepatocellular carcinoma. Oncotarget (2015) 6(4):2538–48. doi: 10.18632/oncotarget.2991 PMC4385869 25576919

[50] Dong T Yan Y Chai H Chen S Xiong X Sun D. Pyruvate kinase M2 affects liver cancer cell behavior through up-regulation of HIF-1alpha and bcl-xL in culture. BioMed Pharmacother (2015) 69:277–84. doi: 10.1016/j.biopha.2014.12.010 25661370

[51] Zeng Z Lan J Lei S Yang Y He Z Xue Y. Simultaneous inhibition of ornithine decarboxylase 1 and pyruvate kinase M2 exerts synergistic effects against hepatocellular carcinoma cells. Onco Targets Ther (2020) 13:11697–709. doi: 10.2147/OTT.S240535 PMC7683510 33244237

[52] Wang Y Yang F Peng Q Mei K He H Yang Q. Long non-coding RNA SNHG1 activates glycolysis to promote hepatocellular cancer progression through the miR-326/PKM2 axis. J Gene Med (2022) 24(8):e3440. doi: 10.1002/jgm.3440 35816558

[53] Sheng T Mao XB Zhang SH. CaMKKbeta regulates proliferation, apoptosis, and glycolysis of hepatocellular carcinoma *via* PI3K/AKT pathway. Ann Palliat Med (2020) 9(6):3857–69. doi: 10.21037/apm-20-1789 33222471

[54] Ye G Qin Y Wang S Pan D Xu S Wu C. Lamc1 promotes the warburg effect in hepatocellular carcinoma cells by regulating PKM2 expression through AKT pathway. Cancer Biol Ther (2019) 20(5):711–9. doi: 10.1080/15384047.2018.1564558 PMC6605989 30755064

[55] Fang Z He L Jia H Huang Q Chen D Zhang Z. The miR-383-LDHA axis regulates cell proliferation, invasion and glycolysis in hepatocellular cancer. Iran J Basic Med Sci (2017) 20(2):187–92. doi: 10.22038/ijbms.2017.8246 PMC5339660 28293396

[56] Wang X Zhang P Deng K. MYC promotes LDHA expression through MicroRNA-122-5p to potentiate glycolysis in hepatocellular carcinoma. Anal Cell Pathol (Amst) (2022) 2022:1435173. doi: 10.1155/2022/1435173 36033372 PMC9410951

[57] Zhang K Mu L Ding MC Xu R Ding ZJ Liang J. NFkappaB mediated elevation of KCNJ11 promotes tumor progression of hepatocellular carcinoma through interaction of lactate dehydrogenase a. Biochem Biophys Res Commun (2018) 495(1):246–53. doi: 10.1016/j.bbrc.2017.11.011 29108994

[58] Zhou Y Huang Y Hu K Zhang Z Yang J Wang Z. HIF1A activates the transcription of lncRNA RAET1K to modulate hypoxia-induced glycolysis in hepatocellular carcinoma cells *via* miR-100-5p. Cell Death Dis (2020) 11(3):176. doi: 10.1038/s41419-020-2366-7 32152275 PMC7062743

[59] Sheng SL Liu JJ Dai YH Sun XG Xiong XP Huang G. Knockdown of lactate dehydrogenase a suppresses tumor growth and metastasis of human hepatocellular carcinoma. FEBS J (2012) 279(20):3898–910. doi: 10.1111/j.1742-4658.2012.08748.x 22897481

[60] Serra M Di Matteo M Serneels J Pal R Cafarello ST Lanza M. Deletion of lactate dehydrogenase-a impairs oncogene-induced mouse hepatocellular carcinoma development. Cell Mol Gastroenterol Hepatol (2022) 14(3):609–24. doi: 10.1016/j.jcmgh.2022.06.003 PMC9307943 35714859

[61] Faloppi L Scartozzi M Bianconi M Svegliati Baroni G Toniutto P Giampieri R. The role of LDH serum levels in predicting global outcome in HCC patients treated with sorafenib: implications for clinical management. BMC Cancer (2014) 14:110. doi: 10.1186/1471-2407-14-110 24552144 PMC3930857

[62] Scartozzi M Faloppi L Bianconi M Giampieri R Maccaroni E Bittoni A. The role of LDH serum levels in predicting global outcome in HCC patients undergoing TACE: implications for clinical management. PloS One (2012) 7(3):e32653. doi: 10.1371/journal.pone.0032653 22461886 PMC3312882

[63] Zhang JP Wang HB Lin YH Xu J Wang J Wang K. Lactate dehydrogenase is an important prognostic indicator for hepatocellular carcinoma after partial hepatectomy. Transl Oncol (2015) 8(6):497–503. doi: 10.1016/j.tranon.2015.11.006 26692531 PMC4700289

[64] Zhou Y Lin F Wan T Chen A Wang H Jiang B. ZEB1 enhances warburg effect to facilitate tumorigenesis and metastasis of HCC by transcriptionally activating PFKM. Theranostics (2021) 11(12):5926–38. doi: 10.7150/thno.56490 PMC8058737 33897890

[65] Zhang D Li Z Li T Luo D Feng X Liu Y. miR-517a promotes warburg effect in HCC by directly targeting FBP1. Onco Targets Ther (2018) 11:8025–32. doi: 10.2147/OTT.S172084 PMC6239112 30519044

[66] Zuo Q He J Zhang S Wang H Jin G Jin H. PPARgamma coactivator-1alpha suppresses metastasis of hepatocellular carcinoma by inhibiting warburg effect by PPARgamma-dependent WNT/beta-Catenin/Pyruvate dehydrogenase kinase isozyme 1 axis. Hepatology (2021) 73(2):644–60. doi: 10.1002/hep.31280 32298475

[67] Sun S Li H Chen J Qian Q. Lactic acid: No longer an inert and end-product of glycolysis. Physiol (Bethesda) (2017) 32(6):453–63. doi: 10.1152/physiol.00016.2017 29021365

[68] Contreras-Baeza Y Sandoval PY Alarcon R Galaz A Cortes-Molina F Alegria K. Monocarboxylate transporter 4 (MCT4) is a high affinity transporter capable of exporting lactate in high-lactate microenvironments. J Biol Chem (2019) 294(52):20135–47. doi: 10.1074/jbc.RA119.009093 PMC6937558 31719150

[69] Halestrap AP. Monocarboxylic acid transport. Compr Physiol (2013) 3(4):1611–43. doi: 10.1002/cphy.c130008 24265240

[70] Gao HJ Zhao MC Zhang YJ Zhou DS Xu L Li GB. Monocarboxylate transporter 4 predicts poor prognosis in hepatocellular carcinoma and is associated with cell proliferation and migration. J Cancer Res Clin Oncol (2015) 141(7):1151–62. doi: 10.1007/s00432-014-1888-8 PMC11823669 25446815

[71] Huang Q Li J Xing J Li W Li H Ke X. CD147 promotes reprogramming of glucose metabolism and cell proliferation in HCC cells by inhibiting the p53-dependent signaling pathway. J Hepatol (2014) 61(4):859–66. doi: 10.1016/j.jhep.2014.04.035 24801417

[72] Reuss AM Groos D Ghoochani A Buchfelder M Savaskan N. MCT4 promotes tumor malignancy in F98 glioma cells. J Oncol (2021) 2021:6655529. doi: 10.1155/2021/6655529 33936203 PMC8060090

[73] Yuan C Zhang J Lou J Wang S Jiang Y Wu F. Comprehensive analysis of monocarboxylate transporter 4 (MCT4) expression in breast cancer prognosis and immune infiltration *via* integrated bioinformatics analysis. Bioengineered (2021) 12(1):3850–63. doi: 10.1080/21655979.2021.1951928 PMC8806482 34269158

[74] Eilertsen M Andersen S Al-Saad S Kiselev Y Donnem T Stenvold H. Monocarboxylate transporters 1-4 in NSCLC: MCT1 is an independent prognostic marker for survival. PloS One (2014) 9(9):e105038. doi: 10.1371/journal.pone.0105038 25225794 PMC4165596

[75] Pereira-Vieira J Azevedo-Silva J Preto A Casal M Queiros O. MCT1, MCT4 and CD147 expression and 3-bromopyruvate toxicity in colorectal cancer cells are modulated by the extracellular conditions. Biol Chem (2019) 400(6):787–99. doi: 10.1515/hsz-2018-0411 30699066

[76] Yan P Li YH Tang ZJ Shu X Liu X. High monocarboxylate transporter 4 protein expression in stromal cells predicts adverse survival in gastric cancer. Asian Pac J Cancer Prev (2014) 15(20):8923–9. doi: 10.7314/APJCP.2014.15.20.8923 25374230

[77] Hu Y Zeng F. Expressions of GPR81, MCT1 and MCT4 in squamous carcinoma and their clinical significance. Zhong Nan Da Xue Xue Bao Yi Xue Ban (2018) 43(9):950–6. doi: 10.11817/j.issn.1672-7347.2018.09.004 30333285

[78] Khan A Valli E Lam H Scott DA Murray J Hanssen KM. Targeting metabolic activity in high-risk neuroblastoma through monocarboxylate transporter 1 (MCT1) inhibition. Oncogene (2020) 39(17):3555–70. doi: 10.1038/s41388-020-1235-2 PMC7970707 32123312

[79] Roland CL Arumugam T Deng D Liu SH Philip B Gomez S. Cell surface lactate receptor GPR81 is crucial for cancer cell survival. Cancer Res (2014) 74(18):5301–10. doi: 10.1158/0008-5472.CAN-14-0319 PMC4167222 24928781

[80] Longhitano L Forte S Orlando L Grasso S Barbato A Vicario N. The crosstalk between GPR81/IGFBP6 promotes breast cancer progression by modulating lactate metabolism and oxidative stress. Antioxidants (Basel) (2022) 11(2):275. doi: 10.3390/antiox11020275 35204157 PMC8868469

[81] Lee YJ Shin KJ Park SA Park KS Park S Heo K. G-Protein-coupled receptor 81 promotes a malignant phenotype in breast cancer through angiogenic factor secretion. Oncotarget (2016) 7(43):70898–911. doi: 10.18632/oncotarget.12286 PMC5342597 27765922

[82] Zan X Fan K Chen K Zhi Y Li L Yang Y. Activation of GPR81 aggravates remote organ injury during hepatic ischemia-reperfusion injury. Transplant Proc (2022) 54(7):1992–7. doi: 10.1016/j.transproceed.2022.04.024 35902290

[83] Kes MMG Van den Bossche J Griffioen AW Huijbers EJM. Oncometabolites lactate and succinate drive pro-angiogenic macrophage response in tumors. Biochim Biophys Acta Rev Cancer (2020) 1874(2):188427. doi: 10.1016/j.bbcan.2020.188427 32961257

[84] Hui S Ghergurovich JM Morscher RJ Jang C Teng X Lu W. Glucose feeds the TCA cycle *via* circulating lactate. Nature (2017) 551(7678):115–8. doi: 10.1038/nature24057 PMC5898814 29045397

[85] Rabinowitz JD Enerback S. Lactate: the ugly duckling of energy metabolism. Nat Metab (2020) 2(7):566–71. doi: 10.1038/s42255-020-0243-4 PMC7983055 32694798

[86] Morrot A da Fonseca LM Salustiano EJ Gentile LB Conde L Filardy AA. Metabolic symbiosis and immunomodulation: How tumor cell-derived lactate may disturb innate and adaptive immune responses. Front Oncol (2018) 8:81. doi: 10.3389/fonc.2018.00081 29629338 PMC5876249

[87] Kim JW Tchernyshyov I Semenza GL Dang CV. HIF-1-mediated expression of pyruvate dehydrogenase kinase: a metabolic switch required for cellular adaptation to hypoxia. Cell Metab (2006) 3(3):177–85. doi: 10.1016/j.cmet.2006.02.002 16517405

[88] Shim H Dolde C Lewis BC Wu CS Dang G Jungmann RA. C-myc transactivation of LDH-a: implications for tumor metabolism and growth. Proc Natl Acad Sci U S A. (1997) 94(13):6658–63. doi: 10.1073/pnas.94.13.6658 PMC21214 9192621

[89] van Hall G. Lactate kinetics in human tissues at rest and during exercise. Acta Physiol (Oxf) (2010) 199(4):499–508. doi: 10.1111/j.1748-1716.2010.02122.x 20345411

[90] Wang T Chen K Yao W Zheng R He Q Xia J. Acetylation of lactate dehydrogenase b drives NAFLD progression by impairing lactate clearance. J Hepatol (2021) 74(5):1038–52. doi: 10.1016/j.jhep.2020.11.028 33248168

[91] Kouzarides T. Chromatin modifications and their function. Cell (2007) 128(4):693–705. doi: 10.1016/j.cell.2007.02.005 17320507

[92] Sabari BR Zhang D Allis CD Zhao Y. Metabolic regulation of gene expression through histone acylations. Nat Rev Mol Cell Biol (2017) 18(2):90–101. doi: 10.1038/nrm.2016.140 27924077 PMC5320945

[93] Dichtl S Lindenthal L Zeitler L Behnke K Schlosser D Strobl B. Lactate and IL6 define separable paths of inflammatory metabolic adaptation. Sci Adv (2021) 7(26):eabg3505. doi: 10.1126/sciadv.abg3505 34162546 PMC8221612

[94] Dai X Lv X Thompson EW Ostrikov KK. Histone lactylation: epigenetic mark of glycolytic switch. Trends Genet (2022) 38(2):124–7. doi: 10.1016/j.tig.2021.09.009 34627643

[95] Gaffney DO Jennings EQ Anderson CC Marentette JO Shi T Schou Oxvig AM. Non-enzymatic lysine lactoylation of glycolytic enzymes. Cell Chem Biol (2020) 27(2):206–13 e6. doi: 10.1016/j.chembiol.2019.11.005 31767537 PMC7395678

[96] Moreno-Yruela C Zhang D Wei W Baek M Liu W Gao J. Class I histone deacetylases (HDAC1-3) are histone lysine delactylases. Sci Adv (2022) 8(3):eabi6696. doi: 10.1126/sciadv.abi6696 35044827 PMC8769552

[97] Dai SK Liu PP Li X Jiao LF Teng ZQ Liu CM. Dynamic profiling and functional interpretation of histone lysine crotonylation and lactylation during neural development. Development (2022) 149(14):dev200049. doi: 10.1242/dev.200049 35735108

[98] Sun Y Chen Y Xu Y Zhang Y Lu M Li M. Genetic encoding of epsilon-N-L-lactyllysine for detecting delactylase activity in living cells. Chem Commun (Camb) (2022) 58(61):8544–7. doi: 10.1039/D2CC02643K 35815577

[99] Galle E Wong CW Ghosh A Desgeorges T Melrose K Hinte LC. H3K18 lactylation marks tissue-specific active enhancers. Genome Biol (2022) 23(1):207. doi: 10.1186/s13059-022-02775-y 36192798 PMC9531456

[100] Irizarry-Caro RA McDaniel MM Overcast GR Jain VG Troutman TD Pasare C. TLR signaling adapter BCAP regulates inflammatory to reparatory macrophage transition by promoting histone lactylation. Proc Natl Acad Sci U S A. (2020) 117(48):30628–38. doi: 10.1073/pnas.2009778117 PMC7720107 33199625

[101] Cui H Xie N Banerjee S Ge J Jiang D Dey T. Lung myofibroblasts promote macrophage profibrotic activity through lactate-induced histone lactylation. Am J Respir Cell Mol Biol (2021) 64(1):115–25. doi: 10.1165/rcmb.2020-0360OC PMC7780997 33074715

[102] Chu X Di C Chang P Li L Feng Z Xiao S. Lactylated histone H3K18 as a potential biomarker for the diagnosis and predicting the severity of septic shock. Front Immunol (2021) 12:786666. doi: 10.3389/fimmu.2021.786666 35069560 PMC8773995

[103] Yang K Fan M Wang X Xu J Wang Y Tu F. Lactate promotes macrophage HMGB1 lactylation, acetylation, and exosomal release in polymicrobial sepsis. Cell Death Differ (2022) 29(1):133–46. doi: 10.1038/s41418-021-00841-9 PMC8738735 34363018

[104] Li L Chen K Wang T Wu Y Xing G Chen M. Glis1 facilitates induction of pluripotency *via* an epigenome-metabolome-epigenome signalling cascade. Nat Metab (2020) 2(9):882–92. doi: 10.1038/s42255-020-0267-9 32839595

[105] Hagihara H Shoji H Otabi H Toyoda A Katoh K Namihira M. Protein lactylation induced by neural excitation. Cell Rep (2021) 37(2):109820. doi: 10.1016/j.celrep.2021.109820 34644564

[106] Hanahan D Weinberg RA. Hallmarks of cancer: the next generation. Cell (2011) 144(5):646–74. doi: 10.1016/j.cell.2011.02.013 21376230

[107] Yu J Chai P Xie M Ge S Ruan J Fan X. Histone lactylation drives oncogenesis by facilitating m(6)A reader protein YTHDF2 expression in ocular melanoma. Genome Biol (2021) 22(1):85. doi: 10.1186/s13059-021-02308-z 33726814 PMC7962360

[108] Xiong J He J Zhu J Pan J Liao W Ye H. Lactylation-driven METTL3-mediated RNA m(6)A modification promotes immunosuppression of tumor-infiltrating myeloid cells. Mol Cell (2022) 82(9):1660–77 e10. doi: 10.1016/j.molcel.2022.02.033 35320754

[109] Jiang J Huang D Jiang Y Hou J Tian M Li J. Lactate modulates cellular metabolism through histone lactylation-mediated gene expression in non-small cell lung cancer. Front Oncol (2021) 11:647559. doi: 10.3389/fonc.2021.647559 34150616 PMC8208031

[110] Yang J Luo L Zhao C Li X Wang Z Zeng Z. A positive feedback loop between inactive VHL-triggered histone lactylation and PDGFRbeta signaling drives clear cell renal cell carcinoma progression. Int J Biol Sci (2022) 18(8):3470–83. doi: 10.7150/ijbs.73398 PMC9134910 35637958

[111] Gu J Zhou J Chen Q Xu X Gao J Li X. Tumor metabolite lactate promotes tumorigenesis by modulating MOESIN lactylation and enhancing TGF-beta signaling in regulatory T cells. Cell Rep (2022) 39(12):110986. doi: 10.1016/j.celrep.2022.110986 35732125

[112] Pan L Feng F Wu J Fan S Han J Wang S. Demethylzeylasteral targets lactate by inhibiting histone lactylation to suppress the tumorigenicity of liver cancer stem cells. Pharmacol Res (2022) 181:106270. doi: 10.1016/j.phrs.2022.106270 35605812

[113] Ferriero R Nusco E De Cegli R Carissimo A Manco G Brunetti-Pierri N. Pyruvate dehydrogenase complex and lactate dehydrogenase are targets for therapy of acute liver failure. J Hepatol (2018) 69(2):325–35. doi: 10.1016/j.jhep.2018.03.016 PMC6057136 29580866

[114] Varner EL Trefely S Bartee D von Krusenstiern E Izzo L Bekeova C. Quantification of lactoyl-CoA (lactyl-CoA) by liquid chromatography mass spectrometry in mammalian cells and tissues. Open Biol (2020) 10(9):200187. doi: 10.1098/rsob.200187 32961073 PMC7536085

[115] Teilhet C Morvan D Joubert-Zakeyh J Biesse AS Pereira B Massoulier S. Specificities of human hepatocellular carcinoma developed on non-alcoholic fatty liver disease in absence of cirrhosis revealed by tissue extracts (1)H-NMR spectroscopy. Metabolites (2017) 7(4):49. doi: 10.3390/metabo7040049 28937622 PMC5746729

[116] Chen Y Zhou J Li J Feng J Chen Z Wang X. Plasma metabolomic analysis of human hepatocellular carcinoma: Diagnostic and therapeutic study. Oncotarget (2016) 7(30):47332–42. doi: 10.18632/oncotarget.10119 PMC5216945 27322079

[117] Fischer K Hoffmann P Voelkl S Meidenbauer N Ammer J Edinger M. Inhibitory effect of tumor cell-derived lactic acid on human T cells. Blood (2007) 109(9):3812–9. doi: 10.1182/blood-2006-07-035972 17255361

[118] Quinn WJ 3rd Jiao J TeSlaa T Stadanlick J Wang Z Wang L. Lactate limits T cell proliferation *via* the NAD(H) redox state. Cell Rep (2020) 33(11):108500. doi: 10.1016/j.celrep.2020.108500 33326785 PMC7830708

[119] Brand A Singer K Koehl GE Kolitzus M Schoenhammer G Thiel A. LDHA-associated lactic acid production blunts tumor immunosurveillance by T and NK cells. Cell Metab (2016) 24(5):657–71. doi: 10.1016/j.cmet.2016.08.011 27641098

[120] Husain Z Huang Y Seth P Sukhatme VP. Tumor-derived lactate modifies antitumor immune response: effect on myeloid-derived suppressor cells and NK cells. J Immunol (2013) 191(3):1486–95. doi: 10.4049/jimmunol.1202702 23817426

[121] Kumar A Pyaram K Yarosz EL Hong H Lyssiotis CA Giri S. Enhanced oxidative phosphorylation in NKT cells is essential for their survival and function. Proc Natl Acad Sci U S A. (2019) 116(15):7439–48. doi: 10.1073/pnas.1901376116 PMC6462103 30910955

[122] Mehla K Singh PK. Metabolic regulation of macrophage polarization in cancer. Trends Cancer (2019) 5(12):822–34. doi: 10.1016/j.trecan.2019.10.007 PMC7187927 31813459

[123] Wynn TA Chawla A Pollard JW. Macrophage biology in development, homeostasis and disease. Nature (2013) 496(7446):445–55. doi: 10.1038/nature12034 PMC3725458 23619691

[124] Bohn T Rapp S Luther N Klein M Bruehl TJ Kojima N. Tumor immunoevasion *via* acidosis-dependent induction of regulatory tumor-associated macrophages. Nat Immunol (2018) 19(12):1319–29. doi: 10.1038/s41590-018-0226-8 30397348

[125] Colegio OR Chu NQ Szabo AL Chu T Rhebergen AM Jairam V. Functional polarization of tumour-associated macrophages by tumour-derived lactic acid. Nature (2014) 513(7519):559–63. doi: 10.1038/nature13490 PMC4301845 25043024

[126] Gabrilovich DI Ostrand-Rosenberg S Bronte V. Coordinated regulation of myeloid cells by tumours. Nat Rev Immunol (2012) 12(4):253–68. doi: 10.1038/nri3175 PMC3587148 22437938

[127] Yang X Lu Y Hang J Zhang J Zhang T Huo Y. Lactate-modulated immunosuppression of myeloid-derived suppressor cells contributes to the radioresistance of pancreatic cancer. Cancer Immunol Res (2020) 8(11):1440–51. doi: 10.1158/2326-6066.CIR-20-0111 32917658

[128] Watson MJ Vignali PDA Mullett SJ Overacre-Delgoffe AE Peralta RM Grebinoski S. Metabolic support of tumour-infiltrating regulatory T cells by lactic acid. Nature (2021) 591(7851):645–51. doi: 10.1038/s41586-020-03045-2 PMC7990682 33589820

[129] Multhoff G Vaupel P. Lactate-avid regulatory T cells: metabolic plasticity controls immunosuppression in tumour microenvironment. Signal Transduct Target Ther (2021) 6(1):171. doi: 10.1038/s41392-021-00598-0 33931598 PMC8087677

[130] Moore EK Strazza M Mor A. Combination approaches to target PD-1 signaling in cancer. Front Immunol (2022) 13:927265. doi: 10.3389/fimmu.2022.927265 35911672 PMC9330480

[131] Hasim MS Marotel M Hodgins JJ Vulpis E Makinson OJ Asif S. When killers become thieves: Trogocytosed PD-1 inhibits NK cells in cancer. Sci Adv (2022) 8(15):eabj3286. doi: 10.1126/sciadv.abj3286 35417234 PMC9007500

[132] Loi S Dushyanthen S Beavis PA Salgado R Denkert C Savas P. RAS/MAPK activation is associated with reduced tumor-infiltrating lymphocytes in triple-negative breast cancer: Therapeutic cooperation between MEK and PD-1/PD-L1 immune checkpoint inhibitors. Clin Cancer Res (2016) 22(6):1499–509. doi: 10.1158/1078-0432.CCR-15-1125 PMC4794351 26515496

[133] Park BV Freeman ZT Ghasemzadeh A Chattergoon MA Rutebemberwa A Steigner J. TGFbeta1-mediated SMAD3 enhances PD-1 expression on antigen-specific T cells in cancer. Cancer Discovery (2016) 6(12):1366–81. doi: 10.1158/2159-8290.CD-15-1347 PMC5295786 27683557

[134] Wei H Xie A Li J Fang C Liu L Xing J. PD-1(+) CD4 T cell immune response is mediated by HIF-1alpha/NFATc1 pathway after p. yoelii infection. Front Immunol (2022) 13:942862. doi: 10.3389/fimmu.2022.942862 36091043 PMC9449323

[135] Man K Gabriel SS Liao Y Gloury R Preston S Henstridge DC. Transcription factor IRF4 promotes CD8(+) T cell exhaustion and limits the development of memory-like T cells during chronic infection. Immunity (2017) 47(6):1129–41 e5. doi: 10.1016/j.immuni.2017.11.021 29246443

[136] Lu P Youngblood BA Austin JW Mohammed AU Butler R Ahmed R. Blimp-1 represses CD8 T cell expression of PD-1 using a feed-forward transcriptional circuit during acute viral infection. J Exp Med (2014) 211(3):515–27. doi: 10.1084/jem.20130208 PMC3949569 24590765

[137] Terawaki S Chikuma S Shibayama S Hayashi T Yoshida T Okazaki T. IFN-alpha directly promotes programmed cell death-1 transcription and limits the duration of T cell-mediated immunity. J Immunol (2011) 186(5):2772–9. doi: 10.4049/jimmunol.1003208 21263073

[138] Chen J Sun HW Yang YY Chen HT Yu XJ Wu WC. Reprogramming immunosuppressive myeloid cells by activated T cells promotes the response to anti-PD-1 therapy in colorectal cancer. Signal Transduct Target Ther (2021) 6(1):4. doi: 10.1038/s41392-020-00377-3 33414378 PMC7791142

[139] Cho HY Lee SW Seo SK Choi IW Choi I Lee SW. Interferon-sensitive response element (ISRE) is mainly responsible for IFN-alpha-induced upregulation of programmed death-1 (PD-1) in macrophages. Biochim Biophys Acta (2008) 1779(12):811–9. doi: 10.1016/j.bbagrm.2008.08.003 18771758

[140] Kinter AL Godbout EJ McNally JP Sereti I Roby GA O'Shea MA. The common gamma-chain cytokines IL-2, IL-7, IL-15, and IL-21 induce the expression of programmed death-1 and its ligands. J Immunol (2008) 181(10):6738–46. doi: 10.4049/jimmunol.181.10.6738 18981091

[141] Munari E Mariotti FR Quatrini L Bertoglio P Tumino N Vacca P. PD-1/PD-L1 in cancer: Pathophysiological, diagnostic and therapeutic aspects. Int J Mol Sci (2021) 22(10):5123. doi: 10.3390/ijms22105123 34066087 PMC8151504

[142] Zerdes I Matikas A Bergh J Rassidakis GZ Foukakis T. Genetic, transcriptional and post-translational regulation of the programmed death protein ligand 1 in cancer: biology and clinical correlations. Oncogene (2018) 37(34):4639–61. doi: 10.1038/s41388-018-0303-3 PMC6107481 29765155

[143] Wang X Yang L Huang F Zhang Q Liu S Ma L. Inflammatory cytokines IL-17 and TNF-alpha up-regulate PD-L1 expression in human prostate and colon cancer cells. Immunol Lett (2017) 184:7–14. doi: 10.1016/j.imlet.2017.02.006 28223102 PMC5362328

[144] Xu L Chen X Shen M Yang DR Fang L Weng G. Inhibition of IL-6-JAK/Stat3 signaling in castration-resistant prostate cancer cells enhances the NK cell-mediated cytotoxicity *via* alteration of PD-L1/NKG2D ligand levels. Mol Oncol (2018) 12(3):269–86. doi: 10.1002/1878-0261.12135 PMC5830627 28865178

[145] Wei Y Zhao Q Gao Z Lao XM Lin WM Chen DP. The local immune landscape determines tumor PD-L1 heterogeneity and sensitivity to therapy. J Clin Invest (2019) 129(8):3347–60. doi: 10.1172/JCI127726 PMC6668685 31112529

[146] Garcia-Diaz A Shin DS Moreno BH Saco J Escuin-Ordinas H Rodriguez GA. Interferon receptor signaling pathways regulating PD-L1 and PD-L2 expression. Cell Rep (2019) 29(11):3766. doi: 10.1016/j.celrep.2019.11.113 31825850

[147] Yan Y Zheng L Du Q Yan B Geller DA. Interferon regulatory factor 1 (IRF-1) and IRF-2 regulate PD-L1 expression in hepatocellular carcinoma (HCC) cells. Cancer Immunol Immunother (2020) 69(9):1891–903. doi: 10.1007/s00262-020-02586-9 PMC10112362 32377817

[148] Gao Y Yang J Cai Y Fu S Zhang N Fu X. IFN-gamma-mediated inhibition of lung cancer correlates with PD-L1 expression and is regulated by PI3K-AKT signaling. Int J Cancer (2018) 143(4):931–43. doi: 10.1002/ijc.31357 29516506

[149] Antonangeli F Natalini A Garassino MC Sica A Santoni A Di Rosa F. Regulation of PD-L1 expression by NF-kappaB in cancer. Front Immunol (2020) 11:584626. doi: 10.3389/fimmu.2020.584626 33324403 PMC7724774

[150] Asgarova A Asgarov K Godet Y Peixoto P Nadaradjane A Boyer-Guittaut M. PD-L1 expression is regulated by both DNA methylation and NF-kB during EMT signaling in non-small cell lung carcinoma. Oncoimmunology (2018) 7(5):e1423170. doi: 10.1080/2162402X.2017.1423170 29721376 PMC5927541

[151] Cheng Y Li H Deng Y Tai Y Zeng K Zhang Y. Cancer-associated fibroblasts induce PDL1+ neutrophils through the IL6-STAT3 pathway that foster immune suppression in hepatocellular carcinoma. Cell Death Dis (2018) 9(4):422. doi: 10.1038/s41419-018-0458-4 29556041 PMC5859264

[152] Zhang W Liu Y Yan Z Yang H Sun W Yao Y. IL-6 promotes PD-L1 expression in monocytes and macrophages by decreasing protein tyrosine phosphatase receptor type O expression in human hepatocellular carcinoma. J Immunother Cancer (2020) 8(1):e000285. doi: 10.1136/jitc-2019-000285 32581055 PMC7319788

[153] Song TL Nairismagi ML Laurensia Y Lim JQ Tan J Li ZM. Oncogenic activation of the STAT3 pathway drives PD-L1 expression in natural killer/T-cell lymphoma. Blood (2018) 132(11):1146–58. doi: 10.1182/blood-2018-01-829424 PMC6148343 30054295

[154] Stutvoet TS Kol A de Vries EG de Bruyn M Fehrmann RS Terwisscha van Scheltinga AG. MAPK pathway activity plays a key role in PD-L1 expression of lung adenocarcinoma cells. J Pathol (2019) 249(1):52–64. doi: 10.1002/path.5280 30972766 PMC6767771

[155] Peng S Wang R Zhang X Ma Y Zhong L Li K. EGFR-TKI resistance promotes immune escape in lung cancer *via* increased PD-L1 expression. Mol Cancer (2019) 18(1):165. doi: 10.1186/s12943-019-1073-4 31747941 PMC6864970

[156] Guo R Li Y Wang Z Bai H Duan J Wang S. Hypoxia-inducible factor-1alpha and nuclear factor-kappaB play important roles in regulating programmed cell death ligand 1 expression by epidermal growth factor receptor mutants in non-small-cell lung cancer cells. Cancer Sci (2019) 110(5):1665–75. doi: 10.1111/cas.13989 PMC6500984 30844110

[157] Takahashi H Jin C Rajabi H Pitroda S Alam M Ahmad R. MUC1-c activates the TAK1 inflammatory pathway in colon cancer. Oncogene (2015) 34(40):5187–97. doi: 10.1038/onc.2014.442 PMC4530107 25659581

[158] Kim EY Kim A Kim SK Chang YS. MYC expression correlates with PD-L1 expression in non-small cell lung cancer. Lung Cancer (2017) 110:63–7. doi: 10.1016/j.lungcan.2017.06.006 28676221

[159] Han H Jain AD Truica MI Izquierdo-Ferrer J Anker JF Lysy B. Small-molecule MYC inhibitors suppress tumor growth and enhance immunotherapy. Cancer Cell (2019) 36(5):483–97 e15. doi: 10.1016/j.ccell.2019.10.001 31679823 PMC6939458

[160] Zhao Y Wang XX Wu W Long H Huang J Wang Z. EZH2 regulates PD-L1 expression *via* HIF-1alpha in non-small cell lung cancer cells. Biochem Biophys Res Commun (2019) 517(2):201–9. doi: 10.1016/j.bbrc.2019.07.039 31331645

[161] Walmsley SR Print C Farahi N Peyssonnaux C Johnson RS Cramer T. Hypoxia-induced neutrophil survival is mediated by HIF-1alpha-dependent NF-kappaB activity. J Exp Med (2005) 201(1):105–15. doi: 10.1084/jem.20040624 PMC2212759 15630139

[162] Ding XC Wang LL Zhang XD Xu JL Li PF Liang H. The relationship between expression of PD-L1 and HIF-1alpha in glioma cells under hypoxia. J Hematol Oncol (2021) 14(1):92. doi: 10.1186/s13045-021-01102-5 34118979 PMC8199387

[163] Sellam LS Zappasodi R Chettibi F Djennaoui D Yahi-Ait Mesbah N Amir-Tidadini ZC. Silibinin down-regulates PD-L1 expression in nasopharyngeal carcinoma by interfering with tumor cell glycolytic metabolism. Arch Biochem Biophys (2020) 690:108479. doi: 10.1016/j.abb.2020.108479 32679194 PMC8507490

[164] Ruf M Moch H Schraml P. PD-L1 expression is regulated by hypoxia inducible factor in clear cell renal cell carcinoma. Int J Cancer (2016) 139(2):396–403. doi: 10.1002/ijc.30077 26945902

[165] Feng W Xue T Huang S Shi Q Tang C Cui G. HIF-1alpha promotes the migration and invasion of hepatocellular carcinoma cells *via* the IL-8-NF-kappaB axis. Cell Mol Biol Lett (2018) 23:26. doi: 10.1186/s11658-018-0077-1 29881400 PMC5984319

[166] Yoo G Park D Kim Y Chung C. New insights into the clinical implications of yes-associated protein in lung cancer: Roles in drug resistance, tumor immunity, autophagy, and organoid development. Cancers (Basel) (2021) 13(12):3069. doi: 10.3390/cancers13123069 34202980 PMC8234989

[167] Kumagai S Koyama S Itahashi K Tanegashima T Lin YT Togashi Y. Lactic acid promotes PD-1 expression in regulatory T cells in highly glycolytic tumor microenvironments. Cancer Cell (2022) 40(2):201–18 e9. doi: 10.1016/j.ccell.2022.01.001 35090594

[168] Tu CE Hu Y Zhou P Guo X Gu C Zhang Y. Lactate and TGF-beta antagonistically regulate inflammasome activation in the tumor microenvironment. J Cell Physiol (2021) 236(6):4528–37. doi: 10.1002/jcp.30169 33230810

[169] Zhang J Huang F Chen L Li G Lei W Zhao J. Sodium lactate accelerates M2 macrophage polarization and improves cardiac function after myocardial infarction in mice. Cardiovasc Ther (2021) 2021:5530541. doi: 10.1155/2021/5530541 34194542 PMC8203388

[170] Dong Q Zhou C Ren H Zhang Z Cheng F Xiong Z. Lactate-induced MRP1 expression contributes to metabolism-based etoposide resistance in non-small cell lung cancer cells. Cell Commun Signal (2020) 18(1):167. doi: 10.1186/s12964-020-00653-3 33097055 PMC7583203

[171] Li X Zhang Z Zhang Y Cao Y Wei H Wu Z. Upregulation of lactate-inducible snail protein suppresses oncogene-mediated senescence through p16(INK4a) inactivation. J Exp Clin Cancer Res (2018) 37(1):39. doi: 10.1186/s13046-018-0701-y 29482580 PMC5828408

[172] Sarkar S Saha P Seth RK Mondal A Bose D Kimono D. Higher intestinal and circulatory lactate associated NOX2 activation leads to an ectopic fibrotic pathology following microcystin co-exposure in murine fatty liver disease. Comp Biochem Physiol C Toxicol Pharmacol (2020) 238:108854. doi: 10.1016/j.cbpc.2020.108854 32781293 PMC7541568

[173] Tauriello DVF Palomo-Ponce S Stork D Berenguer-Llergo A Badia-Ramentol J Iglesias M. TGFbeta drives immune evasion in genetically reconstituted colon cancer metastasis. Nature (2018) 554(7693):538–43. doi: 10.1038/nature25492 29443964

[174] Mariathasan S Turley SJ Nickles D Castiglioni A Yuen K Wang Y. TGFbeta attenuates tumour response to PD-L1 blockade by contributing to exclusion of T cells. Nature (2018) 554(7693):544–8. doi: 10.1038/nature25501 PMC6028240 29443960

[175] Riemann A Reime S Giesselmann M Thews O. Extracellular acidosis regulates the expression of inflammatory mediators in rat epithelial cells. Adv Exp Med Biol (2020) 1232:277–82. doi: 10.1007/978-3-030-34461-0_35 31893421

[176] Riemann A Reime S Thews O. Tumor acidosis and hypoxia differently modulate the inflammatory program: Measurements *In vitro* and in vivo. Neoplasia (2017) 19(12):1033–42. doi: 10.1016/j.neo.2017.09.005 PMC5695649 29149667

[177] Stone SC Rossetti RAM Alvarez KLF Carvalho JP Margarido PFR Baracat EC. Lactate secreted by cervical cancer cells modulates macrophage phenotype. J Leukoc Biol (2019) 105(5):1041–54. doi: 10.1002/JLB.3A0718-274RR 30811636

[178] Gu J Huang X Zhang Y Bao C Zhou Z Jin J. Cytokine profiles in patients with newly diagnosed multiple myeloma: Survival is associated with IL-6 and IL-17A levels. Cytokine (2021) 138:155358. doi: 10.1016/j.cyto.2020.155358 33183958

[179] Li H Liang Q Wang L. Icaritin inhibits glioblastoma cell viability and glycolysis by blocking the IL-6/Stat3 pathway. J Cell Biochem (2018) 120(5):7257–64. doi: 10.1002/jcb.28000 30390336

[180] Mu X Shi W Xu Y Xu C Zhao T Geng B. Tumor-derived lactate induces M2 macrophage polarization *via* the activation of the ERK/STAT3 signaling pathway in breast cancer. Cell Cycle (2018) 17(4):428–38. doi: 10.1080/15384101.2018.1444305 PMC5927648 29468929

[181] Xie Q Zhu Z He Y Zhang Z Zhang Y Wang Y. A lactate-induced Snail/STAT3 pathway drives GPR81 expression in lung cancer cells. Biochim Biophys Acta Mol Basis Dis (2020) 1866(1):165576. doi: 10.1016/j.bbadis.2019.165576 31666207

[182] Han Q Wang Y Pang M Zhang J. STAT3-blocked whole-cell hepatoma vaccine induces cellular and humoral immune response against HCC. J Exp Clin Cancer Res (2017) 36(1):156. doi: 10.1186/s13046-017-0623-0 29115974 PMC5688805

[183] Huang X Gan G Wang X Xu T Xie W. The HGF-MET axis coordinates liver cancer metabolism and autophagy for chemotherapeutic resistance. Autophagy (2019) 15(7):1258–79. doi: 10.1080/15548627.2019.1580105 PMC6613896 30786811

[184] Apicella M Giannoni E Fiore S Ferrari KJ Fernandez-Perez D Isella C. Increased lactate secretion by cancer cells sustains non-cell-autonomous adaptive resistance to MET and EGFR targeted therapies. Cell Metab (2018) 28(6):848–65 e6. doi: 10.1016/j.cmet.2018.08.006 30174307

[185] Qu Y Dou B Tan H Feng Y Wang N Wang D. Tumor microenvironment-driven non-cell-autonomous resistance to antineoplastic treatment. Mol Cancer (2019) 18(1):69. doi: 10.1186/s12943-019-0992-4 30927928 PMC6441162

[186] Gray AL Coleman DT Shi R Cardelli JA. Monocarboxylate transporter 1 contributes to growth factor-induced tumor cell migration independent of transporter activity. Oncotarget (2016) 7(22):32695–706. doi: 10.18632/oncotarget.9016 PMC5078044 27127175

[187] Daneshmandi S Wegiel B Seth P. Blockade of lactate dehydrogenase-a (LDH-a) improves efficacy of anti-programmed cell death-1 (PD-1) therapy in melanoma. Cancers (Basel) (2019) 11(4):450. doi: 10.3390/cancers11040450 30934955 PMC6521327

[188] Pucino V Certo M Bulusu V Cucchi D Goldmann K Pontarini E. Lactate buildup at the site of chronic inflammation promotes disease by inducing CD4(+) T cell metabolic rewiring. Cell Metab (2019) 30(6):1055–74 e8. doi: 10.1016/j.cmet.2019.10.004 31708446 PMC6899510

[189] Shan T Chen S Chen X Wu T Yang Y Li S. M2−TAM subsets altered by lactic acid promote t−cell apoptosis through the PD−L1/PD−1 pathway. Oncol Rep (2020) 44(5):1885–94. doi: 10.1186/s13045-021-01102-5 PMC7551099 33000216

[190] Peng M Yin N Chhangawala S Xu K Leslie CS Li MO. Aerobic glycolysis promotes T helper 1 cell differentiation through an epigenetic mechanism. Science (2016) 354(6311):481–4. doi: 10.1126/science.aaf6284 PMC5539971 27708054

[191] Lu LG Zhou ZL Wang XY Liu BY Lu JY Liu S. PD-L1 blockade liberates intrinsic antitumourigenic properties of glycolytic macrophages in hepatocellular carcinoma. Gut (2022) 71(12):2551–60. doi: 10.1136/gutjnl-2021-326350 PMC9664131 35173040

[192] Lee DC Sohn HA Park ZY Oh S Kang YK Lee KM. A lactate-induced response to hypoxia. Cell (2015) 161(3):595–609. doi: 10.1016/j.cell.2015.03.011 25892225

[193] de Azevedo RA Shoshan E Whang S Markel G Jaiswal AR Liu A. MIF inhibition as a strategy for overcoming resistance to immune checkpoint blockade therapy in melanoma. Oncoimmunology (2020) 9(1):1846915. doi: 10.1080/2162402X.2020.1846915 33344042 PMC7733907

[194] Brown TP Bhattacharjee P Ramachandran S Sivaprakasam S Ristic B Sikder MOF. The lactate receptor GPR81 promotes breast cancer growth *via* a paracrine mechanism involving antigen-presenting cells in the tumor microenvironment. Oncogene (2020) 39(16):3292–304. doi: 10.1038/s41388-020-1216-5 32071396

[195] Feng J Yang H Zhang Y Wei H Zhu Z Zhu B. Tumor cell-derived lactate induces TAZ-dependent upregulation of PD-L1 through GPR81 in human lung cancer cells. Oncogene (2017) 36(42):5829–39. doi: 10.1038/onc.2017.188 28604752

[196] Chen S Zhou X Yang X Li W Li S Hu Z. Dual blockade of Lactate/GPR81 and PD-1/PD-L1 pathways enhances the anti-tumor effects of metformin. Biomolecules (2021) 11(9):1373. doi: 10.3390/biom11091373 34572586 PMC8466555

[197] Song J Lee K Park SW Chung H Jung D Na YR. Lactic acid upregulates VEGF expression in macrophages and facilitates choroidal neovascularization. Invest Ophthalmol Vis Sci (2018) 59(8):3747–54. doi: 10.1167/iovs.18-23892 30046816

[198] Vegran F Boidot R Michiels C Sonveaux P Feron O. Lactate influx through the endothelial cell monocarboxylate transporter MCT1 supports an NF-kappaB/IL-8 pathway that drives tumor angiogenesis. Cancer Res (2011) 71(7):2550–60. doi: 10.1158/0008-5472.CAN-10-2828 21300765

[199] Shime H Yabu M Akazawa T Kodama K Matsumoto M Seya T. Tumor-secreted lactic acid promotes IL-23/IL-17 proinflammatory pathway. J Immunol (2008) 180(11):7175–83. doi: 10.4049/jimmunol.180.11.7175 18490716

[200] Nie W Yu T Sang Y Gao X. Tumor-promoting effect of IL-23 in mammary cancer mediated by infiltration of M2 macrophages and neutrophils in tumor microenvironment. Biochem Biophys Res Commun (2017) 482(4):1400–6. doi: 10.1016/j.bbrc.2016.12.048 27956175

[201] Sonveaux P Copetti T De Saedeleer CJ Vegran F Verrax J Kennedy KM. Targeting the lactate transporter MCT1 in endothelial cells inhibits lactate-induced HIF-1 activation and tumor angiogenesis. PloS One (2012) 7(3):e33418. doi: 10.1371/journal.pone.0033418 22428047 PMC3302812

[202] Zongyi Y Xiaowu L. Immunotherapy for hepatocellular carcinoma. Cancer Lett (2020) 470:8–17. doi: 10.1016/j.canlet.2019.12.002 31811905

[203] He X Xu C. Immune checkpoint signaling and cancer immunotherapy. Cell Res (2020) 30(8):660–9. doi: 10.1038/s41422-020-0343-4 PMC7395714 32467592

[204] Zhang Y Zhang Z. The history and advances in cancer immunotherapy: understanding the characteristics of tumor-infiltrating immune cells and their therapeutic implications. Cell Mol Immunol (2020) 17(8):807–21. doi: 10.1038/s41423-020-0488-6 PMC7395159 32612154

[205] Okazaki T Honjo T. PD-1 and PD-1 ligands: from discovery to clinical application. Int Immunol (2007) 19(7):813–24. doi: 10.1093/intimm/dxm057 17606980

[206] Liu Y Zheng P. Preserving the CTLA-4 checkpoint for safer and more effective cancer immunotherapy. Trends Pharmacol Sci (2020) 41(1):4–12. doi: 10.1016/j.tips.2019.11.003 31836191 PMC7210725

[207] El-Khoueiry AB Sangro B Yau T Crocenzi TS Kudo M Hsu C. Nivolumab in patients with advanced hepatocellular carcinoma (CheckMate 040): an open-label, non-comparative, phase 1/2 dose escalation and expansion trial. Lancet (2017) 389(10088):2492–502. doi: 10.1016/S0140-6736(17)31046-2 PMC7539326 28434648

[208] Zhu AX Finn RS Edeline J Cattan S Ogasawara S Palmer D. Pembrolizumab in patients with advanced hepatocellular carcinoma previously treated with sorafenib (KEYNOTE-224): a non-randomised, open-label phase 2 trial. Lancet Oncol (2018) 19(7):940–52. doi: 10.1016/S1470-2045(18)30351-6 29875066

[209] Liu X Lu Y Qin S. Atezolizumab and bevacizumab for hepatocellular carcinoma: mechanism, pharmacokinetics and future treatment strategies. Future Oncol (2021) 17(17):2243–56. doi: 10.2217/fon-2020-1290 33663220

[210] Yau T Kang YK Kim TY El-Khoueiry AB Santoro A Sangro B. Efficacy and safety of nivolumab plus ipilimumab in patients with advanced hepatocellular carcinoma previously treated with sorafenib: The CheckMate 040 randomized clinical trial. JAMA Oncol (2020) 6(11):e204564. doi: 10.1001/jamaoncol.2020.4564 33001135 PMC7530824

[211] Sangro B Gomez-Martin C de la Mata M Inarrairaegui M Garralda E Barrera P. A clinical trial of CTLA-4 blockade with tremelimumab in patients with hepatocellular carcinoma and chronic hepatitis c. J Hepatol (2013) 59(1):81–8. doi: 10.1016/j.jhep.2013.02.022 23466307

[212] Reyes R Wani NA Ghoshal K Jacob ST Motiwala T. Sorafenib and 2-deoxyglucose synergistically inhibit proliferation of both sorafenib-sensitive and -resistant HCC cells by inhibiting ATP production. Gene Expr (2017) 17(2):129–40. doi: 10.3727/105221616X693855 PMC5296238 27938509

[213] Tomizawa M Shinozaki F Motoyoshi Y Sugiyama T Yamamoto S Ishige N. 2-deoxyglucose and sorafenib synergistically suppress the proliferation and motility of hepatocellular carcinoma cells. Oncol Lett (2017) 13(2):800–4. doi: 10.3892/ol.2016.5510 PMC5351389 28356961

[214] Tomizawa M Shinozaki F Motoyoshi Y Sugiyama T Yamamoto S Ishige N. Suppressive effects of 3-bromopyruvate on the proliferation and the motility of hepatocellular carcinoma cells. Oncol Rep (2016) 35(1):59–63. doi: 10.3892/or.2015.4370 26530887

[215] Yoo JJ Yu SJ Na J Kim K Cho YY Lee YB. Hexokinase-II inhibition synergistically augments the anti-tumor efficacy of sorafenib in hepatocellular carcinoma. Int J Mol Sci (2019) 20(6):1292. doi: 10.3390/ijms20061292 30875800 PMC6471302

[216] Sun X Sun G Huang Y Hao Y Tang X Zhang N. 3-bromopyruvate regulates the status of glycolysis and BCNU sensitivity in human hepatocellular carcinoma cells. Biochem Pharmacol (2020) 177:113988. doi: 10.1016/j.bcp.2020.113988 32330495

[217] Fiume L Vettraino M Manerba M Di Stefano G. Inhibition of lactic dehydrogenase as a way to increase the anti-proliferative effect of multi-targeted kinase inhibitors. Pharmacol Res (2011) 63(4):328–34. doi: 10.1016/j.phrs.2010.12.005 21168502

[218] Billiard J Dennison JB Briand J Annan RS Chai D Colon M. Quinoline 3-sulfonamides inhibit lactate dehydrogenase a and reverse aerobic glycolysis in cancer cells. Cancer Metab (2013) 1(1):19. doi: 10.1186/2049-3002-1-19 24280423 PMC4178217

[219] Manerba M Di Ianni L Govoni M Roberti M Recanatini M Di Stefano G. LDH inhibition impacts on heat shock response and induces senescence of hepatocellular carcinoma cells. Eur J Pharm Sci (2017) 105:91–8. doi: 10.1016/j.ejps.2017.05.015 28501492

[220] Huang T Feng Q Wang Z Li W Sun Z Wilhelm J. Tumor-targeted inhibition of monocarboxylate transporter 1 improves T-cell immunotherapy of solid tumors. Adv Healthc Mater (2021) 10(4):e2000549. doi: 10.1002/adhm.202000549 32431046 PMC7674253

[221] Jeon JY Lee M Whang SH Kim JW Cho A Yun M. Regulation of acetate utilization by monocarboxylate transporter 1 (MCT1) in hepatocellular carcinoma (HCC). Oncol Res (2018) 26(1):71–81. doi: 10.3727/096504017X14902648894463 28390113 PMC7844556

[222] Quanz M Bender E Kopitz C Grunewald S Schlicker A Schwede W. Preclinical efficacy of the novel monocarboxylate transporter 1 inhibitor BAY-8002 and associated markers of resistance. Mol Cancer Ther (2018) 17(11):2285–96. doi: 10.1158/1535-7163.MCT-17-1253 30115664

[223] Ricotti L Tesei A De Paola F Milandri C Amadori D Frassineti GL. Potentiation of antiproliferative drug activity by lonidamine in hepatocellular carcinoma cells. J Chemother (2003) 15(5):480–7. doi: 10.1179/joc.2003.15.5.480 14598941

[224] Nath K Guo L Nancolas B Nelson DS Shestov AA Lee SC. Mechanism of antineoplastic activity of lonidamine. Biochim Biophys Acta (2016) 1866(2):151–62. doi: 10.1016/j.bbcan.2016.08.001 PMC5138080 27497601

[225] Jiang Z Xiong H Yang S Lu Y Deng Y Yao J. Jet-lagged nanoparticles enhanced immunotherapy efficiency through synergistic reconstruction of tumor microenvironment and normalized tumor vasculature. Adv Healthc Mater (2020) 9(12):e2000075. doi: 10.1002/adhm.202000075 32378352

[226] Fang Y Liu W Tang Z Ji X Zhou Y Song S. Monocarboxylate transporter 4 inhibition potentiates hepatocellular carcinoma immunotherapy through enhancing T cell infiltration and immune attack. Hepatology (2022) 1–15. doi: 10.1002/hep.32348 35043976

[227] Salas M Obando P Ojeda L Ojeda P Perez A Vargas-Uribe M. Resolution of the direct interaction with and inhibition of the human GLUT1 hexose transporter by resveratrol from its effect on glucose accumulation. Am J Physiol Cell Physiol (2013) 305(1):C90–9. doi: 10.1152/ajpcell.00387.2012 23615963

[228] Wu H He L Shi J Hou X Zhang H Zhang X. Resveratrol inhibits VEGF-induced angiogenesis in human endothelial cells associated with suppression of aerobic glycolysis *via* modulation of PKM2 nuclear translocation. Clin Exp Pharmacol Physiol (2018) 45(12):1265–73. doi: 10.1111/1440-1681.13017 30044005

[229] Moreira L Araujo I Costa T Correia-Branco A Faria A Martel F. Quercetin and epigallocatechin gallate inhibit glucose uptake and metabolism by breast cancer cells by an estrogen receptor-independent mechanism. Exp Cell Res (2013) 319(12):1784–95. doi: 10.1016/j.yexcr.2013.05.001 23664836

[230] Brito AF Ribeiro M Abrantes AM Mamede AC Laranjo M Casalta-Lopes JE. New approach for treatment of primary liver tumors: The role of quercetin. Nutr Cancer (2016) 68(2):250–66. doi: 10.1080/01635581.2016.1145245 26943884

[231] Azevedo C Correia-Branco A Araujo JR Guimaraes JT Keating E Martel F. The chemopreventive effect of the dietary compound kaempferol on the MCF-7 human breast cancer cell line is dependent on inhibition of glucose cellular uptake. Nutr Cancer (2015) 67(3):504–13. doi: 10.1080/01635581.2015.1002625 25719685

[232] Gunnink LK Alabi OD Kuiper BD Gunnink SM Schuiteman SJ Strohbehn LE. Curcumin directly inhibits the transport activity of GLUT1. Biochimie (2016) 125:179–85. doi: 10.1016/j.biochi.2016.03.014 PMC5006061 27039889

[233] Ojelabi OA Lloyd KP Simon AH De Zutter JK Carruthers A. WZB117 (2-Fluoro-6-(m-hydroxybenzoyloxy) phenyl m-hydroxybenzoate) inhibits GLUT1-mediated sugar transport by binding reversibly at the exofacial sugar binding site. J Biol Chem (2016) 291(52):26762–72. doi: 10.1074/jbc.M116.759175 PMC5207184 27836974

[234] Liu Y Cao Y Zhang W Bergmeier S Qian Y Akbar H. A small-molecule inhibitor of glucose transporter 1 downregulates glycolysis, induces cell-cycle arrest, and inhibits cancer cell growth *in vitro* and in vivo. Mol Cancer Ther (2012) 11(8):1672–82. doi: 10.1158/1535-7163.MCT-12-0131 22689530

[235] Chan DA Sutphin PD Nguyen P Turcotte S Lai EW Banh A. Targeting GLUT1 and the warburg effect in renal cell carcinoma by chemical synthetic lethality. Sci Transl Med (2011) 3(94):94ra70. doi: 10.1126/scitranslmed.3002394 PMC3683134 21813754

[236] Kraus D Reckenbeil J Veit N Kuerpig S Meisenheimer M Beier I. Targeting glucose transport and the NAD pathway in tumor cells with STF-31: a re-evaluation. Cell Oncol (Dordr) (2018) 41(5):485–94. doi: 10.1007/s13402-018-0385-5 PMC12995222 29949049

[237] Siebeneicher H Cleve A Rehwinkel H Neuhaus R Heisler I Muller T. Identification and optimization of the first highly selective GLUT1 inhibitor BAY-876. ChemMedChem (2016) 11(20):2261–71. doi: 10.1002/cmdc.201600276 PMC5095872 27552707

[238] Kang SA O'Neill DJ Machl AW Lumpkin CJ Galda SN Sengupta S. Discovery of small-molecule selective mTORC1 inhibitors *via* direct inhibition of glucose transporters. Cell Chem Biol (2019) 26(9):1203–13 e13. doi: 10.1016/j.chembiol.2019.05.009 31231029

[239] Pelicano H Martin DS Xu RH Huang P. Glycolysis inhibition for anticancer treatment. Oncogene (2006) 25(34):4633–46. doi: 10.1038/sj.onc.1209597 16892078

[240] Feng Y Xiong Y Qiao T Li X Jia L Han Y. Lactate dehydrogenase a: A key player in carcinogenesis and potential target in cancer therapy. Cancer Med (2018) 7(12):6124–36. doi: 10.1002/cam4.1820 PMC6308051 30403008

[241] Li Y Wang K Zhao E Li B Li S Dong X. Prognostic value of lactate dehydrogenase in second-line immunotherapy for advanced esophageal squamous cell carcinoma. Pathol Oncol Res (2022) 28:1610245. doi: 10.3389/pore.2022.1610245 35721326 PMC9203685

[242] Zhang Z Li Y Yan X Song Q Wang G Hu Y. Pretreatment lactate dehydrogenase may predict outcome of advanced non small-cell lung cancer patients treated with immune checkpoint inhibitors: A meta-analysis. Cancer Med (2019) 8(4):1467–73. doi: 10.1002/cam4.2024 PMC6488146 30848091

[243] Payen VL Mina E Van Hee VF Porporato PE Sonveaux P. Monocarboxylate transporters in cancer. Mol Metab (2020) 33:48–66. doi: 10.1016/j.molmet.2019.07.006 31395464 PMC7056923

[244] Zhou J Shao Q Lu Y Li Y Xu Z Zhou B. Monocarboxylate transporter upregulation in induced regulatory T cells promotes resistance to anti-PD-1 therapy in hepatocellular carcinoma patients. Front Oncol (2022) 12:960066. doi: 10.3389/fonc.2022.960066 35965549 PMC9368998

[245] Zhao Y Li W Li M Hu Y Zhang H Song G. Intracellular pH homeostasis and confers self-regulated apoptosis on hepatocellular carcinoma. Exp Cell Res (2019) 384(1):111591. doi: 10.1016/j.yexcr.2019.111591 31479685

[246] Hua H Kong Q Zhang H Wang J Luo T Jiang Y. Targeting mTOR for cancer therapy. J Hematol Oncol (2019) 12(1):71. doi: 10.1186/s13045-019-0754-1 31277692 PMC6612215

[247] Li H Li X Liu S Guo L Zhang B Zhang J. Programmed cell death-1 (PD-1) checkpoint blockade in combination with a mammalian target of rapamycin inhibitor restrains hepatocellular carcinoma growth induced by hepatoma cell-intrinsic PD-1. Hepatology (2017) 66(6):1920–33. doi: 10.1002/hep.29360 28732118

